# Supplementary material for: Low-carbon pathways for the booming express delivery sector in China
Source: Nat Commun. 2021 Jan 19;12:450. doi: 10.1038/s41467-020-20738-4 (PMC7815875; doi:10.1038/s41467-020-20738-4)
Supplement: Supplementary file 1 — Supplementary Information [file 41467_2020_20738_MOESM1_ESM.pdf]

Supporting Information for:

Low-carbon pathways for the booming express delivery sector in China

Tables and Figures

Supplementary Table 1-11

Supplementary Table 1. AIC coefficient for stepwise regression model 6

Supplementary Table 2 Results of stepwise regression for dependent variable GHG emissions 7

Supplementary Table 3. Justifications for scenario analysis 8

Supplementary Table 4. Fee and time for express delivery in different distance 10

Supplementary Table 5. Delivery cost and potential carbon pricing for each piece of parcel 12

Supplementary Table 6. Accuracy analysis on transportation distance model of intra-city delivery (unit: km) 13

Supplementary Table 7. Data sources related to express deliveries 14

Supplementary Table 8. Summary of field surveys conducted between 2018 and 2019 16

Supplementary Table 9. Type and specifications of deliveries by packaging materials types 17

Supplementary Table 10. Emission factors of transportation modes 18

Supplementary Table 11. Function distribution for a few parameters 19

Supplementary Figures 1 to 38

Supplementary Fig.1 Social retail sales, E-commerce, and online retail sales from 2008 to 2018 in China ...20

Supplementary Fig.2 Total volume of express delivery packages and express revenue from 2008 to 2018 in China 21

Supplementary Fig.3 Full-time staff of e-commerce and online shopping subscribers from 2008 to 2018 in China 22

Supplementary Fig.4 Coupling and coordination of three parts from 2008 to 2018. Three parts include: economic indicators (E-commerce sales, social retail sales, and on-line retail sales), express development indicators (express delivery volume and express income), and social indicators (full-time staff and online shopping phone)....23

Supplementary Fig.5 Quantity of express deliveries (by piece) in different provinces in mainland China (31 provinces and municipality) 25

Supplementary Fig.6 Projection of express receive per person and the express delivery volume in China 26

Supplementary Fig.7 Technology route of this study 27

Supplementary Fig.8 Estimated composition of express deliveries (by package materials type) in China in 2018 (by piece)....28

Supplementary Fig.9 Total GHG emissions estimate from the logistics and transportation of online shopping parcels: divided by types of pracles or packages materials (2018) 29

Supplementary Fig.10 Total GHG emissions from the logistics and transportation of express delivery (intra-cities and inter-cities delivery): (A) by regional centers; (B) By provinces. Each region covers areas within the distribution radius from a regional center (a city) represented by a dot. 30

Supplementary Fig.11 Total GHG emissions from the logistics and transportation of intra-cities express delivery (by regional centers/cities)....31

Supplementary Fig.12 Total GHG emissions from the logistics and transportation of inter-cities express delivery (by regional centers/cities)....32

Supplementary Fig. 13 GHG emissions from the logistics and transportation of intra-cities express delivery (by seven geographical regions) 33

Supplementary Fig.14 GHG emissions from the logistics and transportation of inter-cities express delivery (by seven geographical regions) 34

Supplementary Fig.15 GHG emissions from the logistics and transportation of express delivery (inter-city express delivery, Phase I to V)..35
Supplementary Fig.16 Five transport phases of inter-city express delivery: a case study 36
Supplementary Fig.17 Delivery volume and receive volume of express deliveries for each regional center..37
Supplementary Fig.18 Average distance of phase II and IV of inter-city express delivery 38
Supplementary Fig. 19 Transport distance of phase II and IV (average value) of inter-city express delivery.39
Supplementary Fig.20 Correlation ship analysis between social-economic factors and GHG emissions from the shipment of express delivery of inter-city express delivery (correlation analysis from the total volume). 40
Supplementary Fig.21 Correlation ship analysis between social-economic factors and GHG emissions from the shipment of express delivery of inter-city express delivery (correlation analysis from per piece). ...41
Supplementary Fig.22 Distance of three types of transport type for each piece of parcel of inter-city express delivery..42
Supplementary Fig.23 Proportion of three types of transportation mode for each piece of parcel of inter-city express delivery 43
Supplementary Fig. 24 Correlation ship analysis between social-economic factors and GHG emissions from the shipment of express delivery of inter-city express delivery...44
Supplementary Fig.25 GHG emissions from express deliveries under various scenarios in China. 45
Supplementary Fig. 26 An estimate of the GHG emissions mitigation potentials subject the adjustment of delivery mode for inter-city express delivery service: Tradeoff between Speed (cost) and carbon saving 46
Supplementary Fig.27 Results of questionnaire on carbon labelling of inter-city express delivery................47
Supplementary Fig.28 Goal and systemic boundary definition of this study..49
Supplementary Fig.29 Modeling for three types of transportation phase for intra-city express delivery service-a case study of Shanghai city: (a) land cover and land use of Shanghai city; (b) buildup area of Shanghai city; (c) three types of transport phase and corresponding estimated distances.....................................50
Supplementary Fig.30 The hub and spoke network of inter-city express delivery..51
Supplementary Fig.31 Spatial configuration of the regional centers in the hub and spoke model of inter-city express delivery 52

Supplementary Fig.32 Each center covering relative national cities 53

Supplementary Fig.33 The matrix of transferring ratios among 42 regional centers 54

Supplementary Fig.34 Proportion of three transportation type 55

Supplementary Fig.35 Sensibility analysis of model parameter for GHG emissions....................................56

Supplementary Fig.36 Monte Carlo simulation for the total weight of scrap packaing materials..................57

Supplementary Fig.37 Monte Carlo simulation for GHG emission from the shipment of express delivery: by distribution...58

Supplementary Fig.38 Monte Carlo simulation of GHG emission from the shipment of express delivery of four major package categories 59

1. GHGs quantification of logistics and transportation sector

Several studies suggest that logistics activities and transportation are influential sources of anthropogenic greenhouse gases (GHG)[1,2]. According to the World Economic Forum and Accenture, logistical activity accounts for about 5.5% of the global GHG emissions, with 90% of these GHG stemming from freight transport, and two-third of these transport GHGs emissions are generated by trucks and vans [3]. In the recent years, several organizations have published guidelines on how to quantify GHG emissions from freight transport operations[4,5].

The European Norm EN 16258 'Methodology for calculation and declaration of energy consumption and GHG emissions of transport services (freight and passengers)'[6], which was published in 2012 by the European Committee for Standardization (CEN), is presently the only official international—though European—standard for emission calculation of transportation in supply chains[7,8]. The national standardization bodies of 33 countries are obliged to adopt this norm. The European Norm EN 16258 provided a common methodology for the calculation and declaration of energy consumption and greenhouse gas emissions related to any transport operation. In order to guarantee an accurate, transparent, and comparable quantification of GHGs resulting from supply chain activities, some scholars have made the improvement to enchance the performance of the guidelines [9,10]. In total ,the guideline was proved to offer a pragmatic and scientifically-acceptable approach that allows a wide group of users to prepare standardized, accurate, credible, comparable, and verifiable energy consumption and emission declarations. Therefore, we use the standard EN 16258: 2012, the most acceptable standard globally.

2. Supplementary Tables and Figures

Supplementary Table 1. AIC coefficient for stepwise regression model

Index	AIC								
	All	-RAT	-GPP	-TDPP	-ADPP	-RRAT	-EPP	-DTPP	-RROT
RDPP	-73.602	-75.546	-76.715	-73.075	-74.864	-39.728	-39.982	-18.798	11.994
UR	-74.402	-76.360	-76.195	-77.318	-78.028	-79.948	-81.800	-83.145	-85.141
DVPP	-77.991	-79.943	-81.899	-83.856	-85.551	-86.920	-84.092	-85.214	-86.339
PTI	-75.531	-77.531	-79.369	-81.278	-83.071	-84.898	-86.700	-87.473	**89.072**
RROT	-79.588	-80.633	-82.489	-84.365	-86.272	-87.249	-88.234	**-90.104**	
DTPP	-78.316	-79.874	-81.695	-83.418	-85.275	-87.235	**-88.324**		
EPP	-78.947	-80.885	-82.719	-84.431	-85.879	**-87.294**			
RRAT	-79.579	-80.858	-82.518	-84.393	**-86.310**				
ADPP	-79.303	-81.221	-83.073	**-84.970**					
TDPP	-79.440	-81.387	**-83.316**						
GPP	-79.542	**-81.494**							
RAT	**-79.593**								

(*Note: RDPP: Road transportation distance per piece, UR: Urbanization ratio,DVPP: Delivery volume per person, PTI: Proportion of tertiary industry,RROT: Ratio of road transportation, DTPP: Delivery time per piece, EPP: Express expenses per person, RRAT: Ratio of railway transportation, ADPP: Air transportation distance per piece, TDPP: train transportation distance per piece , GPP:GDP per person, RAT : Ratio of air transportation, Y: GHG emission per piece)*

Here, a stepwise approach for predictor selection was applied, based on theminimization of the Akaike Information Criterion (AIC). Based on the concept of entropy, AIC is used to estimate the complexity of the estimated model and the goodness of the model fitting data.

Supplementary Table 2 Results of stepwise regression for dependent variable GHG emissions

	Estimate	Std.Error	t value	P
Intercept	2.615	0.4638	5.639	1.94E-06
x1	0.0023	0.0013	20.104	<2e-16***
x2	-1.751	0.6781	-2.583	0.0139*
x3	0.002	0.0008	2.333	0.0252*
x4	-1.5590	0.9367	-1.664	0.1045

Note: *, $p<0.05$; **, $p<0.01$; ***, $p<0.001$. x1: Road transportation distance per piece, x2: Urbanization ratio, x3: Delivery volume per person, x4: Proportion of tertiary industry

Supplementary Table 3. Justifications for scenario analysis

Scenario	Background and significance	Relevant measures
S1 (Faster delivery)	S1 and S2 are justified by the Chinese government's plan for the express delivery industry[12] in which diversified modes of express delivery services are encouraged including both faster delivery speed and slower one to serve different consumer demands.	Increasing the proportion of aircraft transportation could increase delivery speed for S1. Specifically, the number of dedicated cargo planes increased from 57 in 2013 to 116 in 2018 in China.[13,14] So far, three express delivery companies have their own airline fleets.[12] It is true that delivery time is becoming shorter and shorter; this is a key indicator to evaluate the quality and competitiveness of an e-commerce company. For example, the average delivery time by 'Tmall' (one of the top e-commerce companies in China) has fallen from 9 days in 2013 to 2.8 days in 2018.[15]
S2 (Slower delivery)		Many deliveries are not urgent, thus do not necessarily need fast delivery. Slower, cheaper, and also less carbon-intensive delivery services are preferred for such demand, such as delivery by railways.[16] Express delivery companies are indeed developing railway-based express delivery services.[17]
S3 (Fuel standard upgrade)	Fuel standard upgrade is a nation-wide policy that affects all transportation-related sectors including the express delivery sector (National Development and Reform Commission (NDRC) Announcement on Oil Products Upgrading-Announcement No.16. [18])	The National Development and Reform Commission (NDRC) in China has issued more stringent standards leading to the implementation of China VI standards (Euro 4) in 2020.
S4 (Less packaging material)	China's State Council issued the "Express Delivery Interim Regulations" in 2019 including a mandate to reduce packaging materials for express deliveries by 15% through means such as improved source control and using environmentally friendly packaging products.	Along with the release of the revised version of the "Packing Standard for Express Service" (GB/T 16606-2018) in 2018 [19] which sets higher environmental standards for packaging products, the State Post Bureau has begun to vigorously promote the implementation

Scenario	Background and significance	Relevant measures
		of the “9571 program*” which targets at reducing the amount of packaging materials.[17] Express delivery companies have invested significantly in environmentally friendly packaging, such as the Alibaba Green Logistics 2020 plan, the Jingdong-Qingliu plan, the Suning-Qingcheng plan, and the SF express-Fengjing plan.[19]
S5 (Logistics optimization)	The transportation and logistics network is one of the most important asset of express delivery companies. Constantly optimizing the network by increasing delivery volume and speed is a major undertaking for the companies to reduce cost and improve custom satisfaction. As a result, it also reduce GHG emissions and other environmental imapcts.[20]	Express delivery companies are constantly optimizing their transportation and logistics networks, by introducing innovative technologies (such as cloud computing, big data, artificial intelligence, and blockchain).[21]

* *The “9571 program” means: the proportion of electronic waybill will account for 95% of the total, over 50% of the parcels will not use external packaging materials for delivering purposes, the reusable transshipment packaging bags will reach 70% of the total, and more than 100,000 express delivery service centers will recycle packaging materials.*

Supplementary Table 4. Fee and time for express delivery in different distance

Original (sending out) cities	Destination (receiving) cities	Distance (km)	Air		Road		Railway	
			time	fee	time	fee	time	fee
Fuzhou	Nanchang	618	30	3.23	60	2.64	96	2.35
Shijiazhuang	Harbin	705	30	3.37	60	2.64	96	2.49
Xiamen	Hefei	806	30	3.37	60	2.64	96	2.49
Wenzhou	Wuhan	895	30	3.23	60	2.64	96	2.35
Taizhou	Qingdao	1001	30	3.23	60	2.64	96	2.35
Zhengzhou	Harbin	1113	30	3.23	60	2.64	96	2.35
Hangzhou	Shenzhen	1202	30	3.37	60	2.64	96	2.49
Shenzhen	Chongqing	1311	30	3.37	60	2.64	96	2.49
Harbin	Beijing	1413	30	3.37	60	2.64	96	2.49
Shanghai	Xi’an	1510	30	3.37	60	2.64	96	2.49
Nanchang	Taiyuan	1600	30	3.37	60	2.64	96	2.49
Nanjing	Shenzhen	1707	30	3.37	60	2.64	96	2.49
Tianjin	Lanzhou	1813	30	3.37	60	2.64	96	2.49
Tianjin	Wenzhou	1902	30	3.37	60	2.64	96	2.49
Nanjing	Changchun	2013	30	3.37	60	2.64	96	2.49
Shanghai	Kunming	2100	30	3.37	60	2.64	96	2.49
Nanning	Shijiazhuang	2208	30	3.37	84	2.64	96	2.49
Guangzhou	Taiyuan	2301	30	3.37	60	2.64	96	2.49
Xi’an	Changchun	2416	30	3.37	60	2.64	96	2.49
Xiamen	Xi’an	2514	30	3.37	60	2.64	96	2.49
Lanzhou	Changchun	2600	30	3.37	84	2.64	96	2.49
Tianjin	Nanning	2722	30	3.37	84	2.64	96	2.49
Jieyang	Hohhot	2806	30	3.37	84	2.64	96	2.49
Huaian	Haikou	2906	30	3.37	84	2.64	96	2.49
Kunming	Tianjin	3019	30	3.37	84	2.64	96	2.49
Shenyang	Nanning	3098	30	3.37	84	2.64	96	2.49
Harbin	Shenzhen	3208	30	3.37	84	2.64	96	2.49
Lhasa	Hohhot	3320	30	3.37	84	2.64	96	2.49
Urumqi	Shenyang	3394	30	3.37	84	2.64	96	2.49
Urumqi	Qingdao	3514	48	3.52	84	2.93	96	
Urumqi	Nanchang	3700	48	3.52	108	2.93	96	
Urumqi	Changsha	3902	48	3.52	108	2.93	96	
Lhasa	Kunming	4100	48	3.52	84	2.93	96	
Kunming	Urumqi	4216	48	3.52	108	2.93	96	

Original (sending out) cities	Destination (receiving) cities	Distance (km)	Air		Road		Railway	
			time	fee	time	fee	time	fee
Lhasa	Ningbo	4297	48	3.52	84	2.93	96	
Urumqi	Changchun	4507	52	4.11	132	3.23	96	
Lhasa	Changchun	4880	52	4.11	132	3.23	96	
Lhasa	Guangzhou	4980	52	4.11	132	3.23	96	
Haikou	Urumqi	5107	52	4.11	132	3.23	96	

Supplementary Table 5. Delivery cost and potential carbon pricing for each piece of parcel

Type of express delivery service	Delivery cost	Carbon pricing*	Fraction**
	USD (on average, per piece)		%
Intra-city delivery	1.39	1.59e-05	0.001%
Inter-city delivery	2.79	0.002	0.072%
Total	2.46	0.0015	0.061%

(**:carbon pricing was sourced form carbon market pricefluctuation in China, **: fraction of total cost*)

Supplementary Table 6. Accuracy analysis on transportation distance model of intra-city delivery (unit: km)

Regions	Reference value	The value by model	Error
China	24.46	23.19	5.19%
Shenzhen	24.70	24.33	1.40%
Guangzhou	28.28	28.87	2.08%
Suzhou	28.53	30.21	5.08%

Note: the reference model of three cities was sourced from the statistical data of the local postal bureau in 2017 or 2018.

With consideration of the differences in geographical location and postal statistics of all cities, the accuracy of the model was evaluated from a national scale. The transportation distance (on average) of a parcel within intra-city express delivery service in each city was calculated by the assessing model (Figure S8). Then the average transportation distance in all Chinese cities was gotten by equation 1. In our study, the average value was 23.2km.

$$Dis_{ave} = \sum_{i=0}^{n} Dis_i * \frac{ExV_i}{ExV_{sum}} \qquad S1$$

where Dis_{ave} is the average transportation distance of intra-city express delivery in China, Dis_i is the averge transportation distance of a parcel within intra-city express delivery service in the city *i*, ExV_i is the express delivery volume of the city *i, ExV_{sum}* is the total express delivery volume in China, and *m* is the cities number of the country.

Meanwhile, the reference value for comparison purpose was the average length of the urban postal delivery route in China, which was collected from the Chinese Statistic Bulletin of Post Business in 2018 (State Post Bureau of China (SPBC)). There were 70,000 postal delivery routes of cities in nationwide, the total length of urban delivery routes (one-way) is 1.72 million kilometers. Therefore, the reference value (on average) was 24.46 km for each city.

The accuracy of the model as shown in Table S4. The error in at national level was 5.19%. Meanwhile, the errors of three major cities were all below 6%. These errors analysis indicated that our model had good performance and reasonable feasibility.

Supplementary Table 7. Data sources related to express deliveries

Important parameters		Formula	Unit	Major Sources
Parcels	Delivery volume	Inter-city express service, $D_{c,y}$.<br>Intra-city express service, $D_{u,y}$.	piece	The volume and distribution ratio of express delivery of inter-city and intra-city was sourced from the postal statistics annual report of each city (Figure S2)
	Package weight	Parcel weight, Wp.<br>Packaging materials weight, Wp_m.<br>Reusable rate of major packaging materials, rt_i	kg	A comprehensive investigation of on package type and weight in a number of distribution centers in various cities between 2018 and 2019. Surveys on parcels weight in Shenzhen, Wuhan and Lanzhou of China, the samples were 5,100 pieces.(Figure S8).
	Package type configuration	The proportion of type i of each parcel (Table S2), pt_i.	%	Field surveys conducted between 2018 and 2019 (Table S6) to determine the types (t) of express delivery considering the diversities of materials (m) and sizes (j) of packages (large, middle, and small), their corresponding weights (Q). The samples were 8,010 pieces.
Logistics & transportation	Modes of transportation	Phase I, V, and Intra-D		The first part was truck, the last-mile transportation was the electric vehicle.
		Phase II and V		The transportation tool was truck (road).
		Phase III: road, air, and train<br>The proportion of transport type (road, air, and railway), Pi.	%	The proportion of transport type (road, air, and railway) was shown in Fig.S34.
		The transferring proportion from origin to destination, OD_{pi}.	%	We gathered a sample size of approximately 20,000 electronic order slips showing delivery origin and destination to determine the transportation mode of parcels shipped.
	Shipment distance	The transportation distance of the Intra-D parcels, Td_{ci}.<br>The last-one-kilometer transportation distance, Dl.	km	Last-mile transport: sourced from the postal statistics annual report of each city (reports).
		The transportation distance of the branch line transportation, Dt_b.	km	Road: acquired by Baidu map(internet).

Important parameters		Formula	Unit	Major Sources
		The distance of the corresponding transport type, Dt_{mi}.	km	Considering the distance of three types of transportation, the transportation distance of road was acquired by Baidu Map, the route distance of airline was acquired by China Southern Airlines, and while transportation distance of train was acquired by http://www.huochepiao.com/.
Other social economic factors	socioeconomic	urbanization ratio (UR)	%	Socioeconomic data were from the Statistical Yearbooks of the local statistical offices in2018.
		GDP	USD	
		tertiary industry proportion (SIP)	%	
	Express statistic factors	personal express delivery expenses	USD	The express delivery volume and income of each city was sourced from local postal bureau in 2018.
		express delivery income	USD	

Supplementary Table 8. Summary of field surveys conducted between 2018 and 2019

Objective	Survey	Samples	Location
Weight of package, Types of packages (8010 pieces)	Shenzhen city (3000 pieces)	2,800 pieces	Shenzhen University
		100 pieces	Houde pinyuan community, Nanshan district
		100 pieces	Xuefu garden community, Nanshan district
	Guangzhou city	320 pieces	Guangzhou University
	Wuhan city (1760 pieces)	580 pieces	Wuhan University of Technology
		280 pieces	Pengxiang garden community, Jiangxia district
		200 pieces	Foao Junxian Yaju community, Jiangxia district
		500 pieces	Dangdai international garden community, Jiangxia district
		200 pieces	Gao community, Jiangxia district
	Pingdingshan city	440 pieces	Henan University of Urban Construction
	Shanghai city (370 pieces)	150 pieces	Shanghai Polytechnic University
		220 pieces	Tongji University
	Hangzhou city	230 pieces	Hangzhou Dianzi University
	Ningbo city	410 pieces	Ningbo University
	Fuzhou city	240 pieces	Fujian University of Technology
	Beijing city	470 pieces	Peking University
	Tianjin city	280 pieces	Nankai University
	Taiyuan city	150 pieces	North University of China
	Lanzhou city (340 pieces)	180 pieces	Northwest Normal University
		160 pieces	LanZhou University of Finance and Economics
Packaging materials types	Shenzhen city	300 packages	Express delivery distribution & receiving centers (Cainiao Yizhan, Shunfeng express sites)
	Wuhan city	250 packages	
Express waybills*	42 regional centers	500 pieces each center	

*Note: *, Tracking the logistic & transportation of express deliveries (transportation mode and distance).*

Supplementary Table 9. Type and specifications of deliveries by packaging materials types

Type of package	Size	Specifications(mm)		Weight
		min	max	(kg)
Corrugated box	Oversize	530*320*230	≥700*400*320	4.04
	Large	360*300*250	530*320*230	2.37
	Medium	300*250*200	360*300*250	1.27
	Small	≤200*180*100	250*200*180	0.28
	Plastic bag carton(Large)	300*350*200	360*300*250	0.70
	Plastic bag carton(Small)	200*180*100	250*200*180	0.26
Plastic bag	Variegated plastic bag (Large)	≥400*320		0.76
	Variegated plastic bag (Small)	300*250	400*320	0.25
	Plastic (black & gray) bag (Large)	≥400*320		0.81
	Plastic (black & gray) bag (Small)	300*250	400*320	0.22
	Plastic (white & pure) bag (Large)	≥400*320		0.77
	Plastic (white & pure) bag (small)	300*250	400*320	0.25
Other packages	Woven bags(Large)	≥540*420		4.84
	Woven bags(Small)	300*200	500*420	1.82
	Air bubble bags (Large)	≥400*320		0.79
	Air bubble bags (Small)	300*250	400*320	0.07
	Foam materials(Large)	360*300*250	530*320*230	4.40
	Foam materials(Small)	300*250*200	360*300*250	1.63
File envelopes	File envelopes (Large)	280*180	240*160	2.39
	File envelopes (Small)	240*160	280*180	0.49
	Paper tube (Large)	≥600	≥600	0.07
	Paper tube (Small)	300	300	0.06

Supplementary Table 10. Emission factors of transportation modes

Tools	Value	Unit	Data source
Electric vehicle	0.113	kg CO_2e/tkm	eBalance
8 metric ton freight truck	0.103	kg CO_2e/tkm	eBalance
16-32 metric tons freight trucks	0.167	kg CO_2e/tkm	Ecoinvent
Electric freight train	0.057	kg CO_2e/tkm	Ecoinvent
Freight aircraft	1.185	kg CO_2e/tkm	Ecoinvent

With consideration of the complexity of the model and combined with our investigations, the proportion of transportation mode of the mainline, the package weight and the ratio of four main package categories are the major parameters. Based on the data distribution characteristics in the sample, the distribution for package weight represents normal distribution, while the distribution for other parameters represents uniform distribution.

Supplementary Table 11. Function distribution for a few parameters

Parameters	Distribution	Description
The proportion of airline in mainline transportation	uniform distribution	The maximum is 0.90, while the minimum is 0.78.
The proportion of truck in mainline transportation	uniform distribution	The maximum is 0.15, while the minimum is 0.05.
Package weight of the medium corrugated box	normal distribution	The mean is 1.59, and the standard deviation is 1.3.
Package weight of the large corrugated box	normal distribution	The mean is 3.04, and the standard deviation is 2.80.
Package weight of the large plastic (black & gray) bag	normal distribution	The mean is 0.80, and the standard deviation is 0.91.
The ratio of corrugated box	uniform distribution	The maximum is 0.60, while the minimum is 0.48.
The ratio of plastic bag	uniform distribution	The maximum is 0.38, while the minimum is 0.28.
The ratio of file envelopes	uniform distribution	The maximum is 0.10, while the minimum is 0.05.

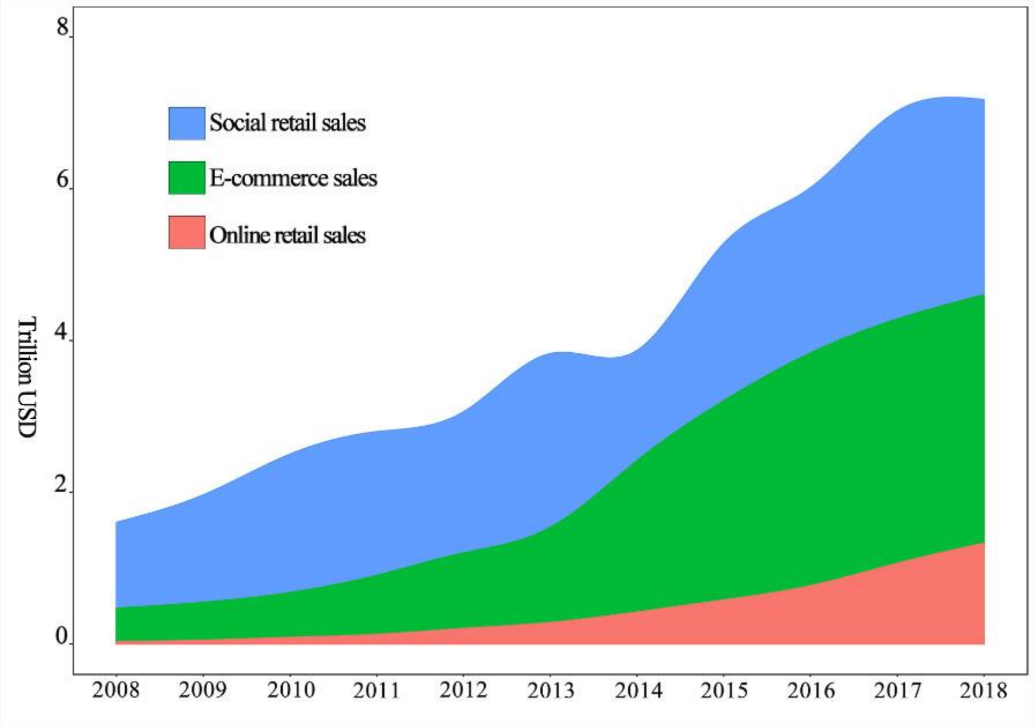

Supplementary Fig.1 Social retail sales, E-commerce, and online retail sales from 2008 to 2018 in China
(*Online retailing is customer-to-customer (C2C), which is a part of E-commerce. Specifically, E-commerce is divided into B2B, B2C, C2C and so on.***)**

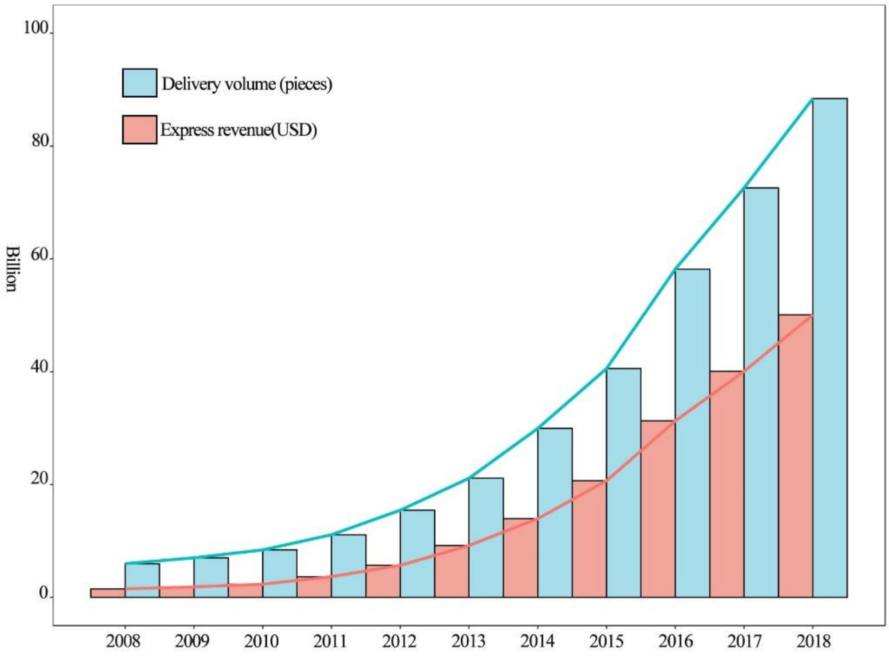

Supplementary Fig.2 Total volume of express delivery packages and express revenue from 2008 to 2018 in China

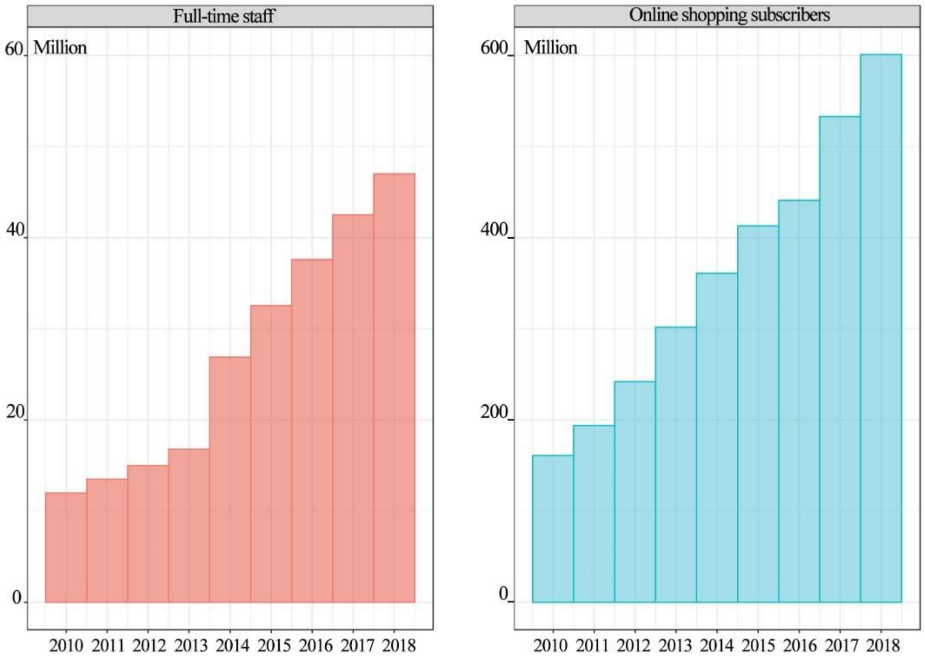

Supplementary Fig.3 Full-time staff of e-commerce and online shopping subscribers from 2008 to 2018 in China

(*Data source: The report of e-commerce in China***)**

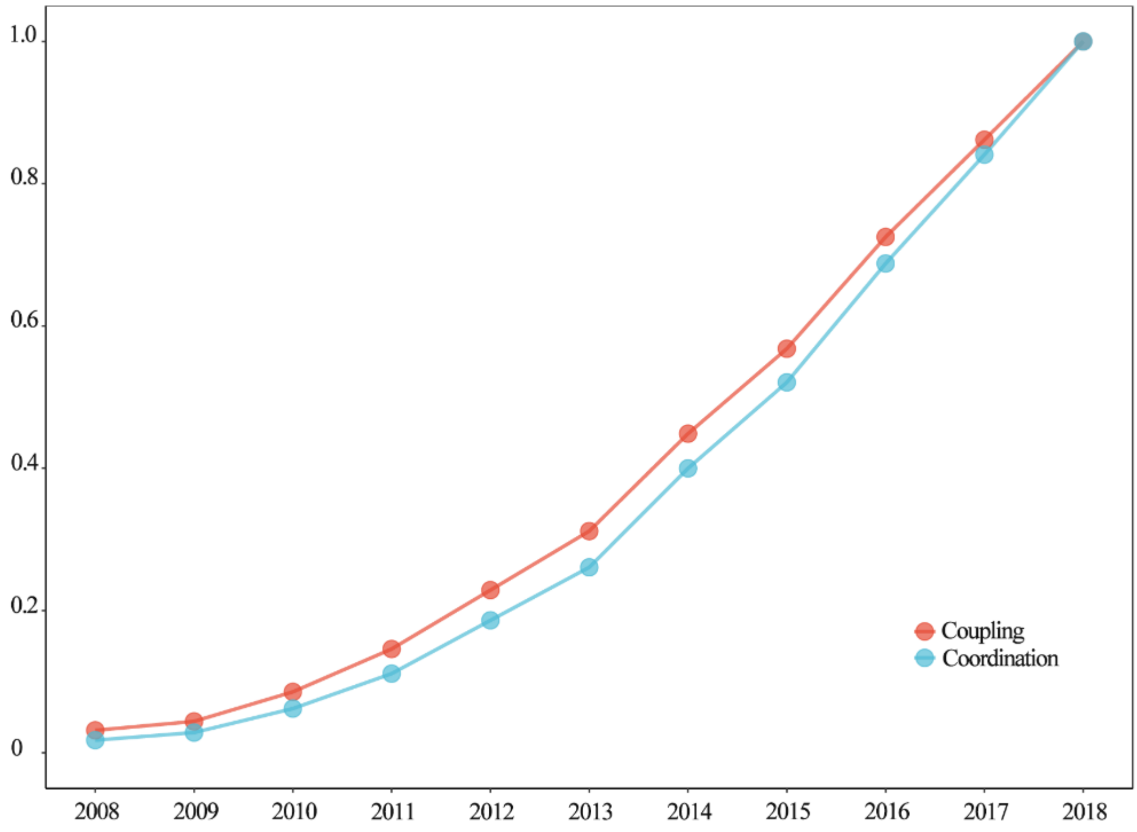

Supplementary Fig.4 Coupling and coordination of three parts from 2008 to 2018. Three parts include: economic indicators (E-commerce sales, social retail sales, and on-line retail sales), express development indicators (express delivery volume and express income), and social indicators (full-time staff and online shopping phone)

Coupling degree

Coupling degree was used to analyze the coordination degree of two systems development. Data on sales indicators (e-commerce sales, social retail sales, and online retail sales), express delivery indicators (express delivery volume and express income), and social indicators (full-time staff and online shopping subscribers) were collected from the China Statistical Yearbooks (2008–2018). Here, we standardized the data using Eq.S2 and eliminated the influence of dimension and magnitude. Then weighting formula was applied to calculate the comprehensive value of each part (S3). Finally, the coupling degree model (S4) was utilized to calculate the coupling degree and coordinationof three parts.

$$X'_{ij} = \left(x_{ij} - \min\{X_j\}\right) / \left(\max\{X_j\} - \min\{X_j\}\right) \qquad \text{S2}$$

where Xij represents the value of indicator j in year i, and max{Xj} and min{Xj} indicate the minimum and maximum values of indicator j among all years.

$$Y_a = \sum_{i=1}^{n} x_{ij} w_j \qquad \text{S3}$$

whereYi represents the value of each part, wj represents the weight of indicator j in part a.

$$C=\left\{\frac{y_a*y_b*y_c}{\left[\frac{y_a+y_b+y_c}{3}\right]^3}\right\}^{1/3}$$ S4

The coupling degree C ranges from 0 to 1. The C is close to 1 indicate that the greater degree of coupling among the systems. However, the C is close to 0 represent the system is disorder and the development is limited.

$$D=\sqrt{C*T},T=\partial*y_a+\beta*y_b+\chi*y_c$$ S5

where C is the coupling degree, D is the coupling coordination, and T is the comprehensive evaluation index of the coupling coordination development level; respectively, the weight of each subsystem.

The coupling degree and coordinate were increased from 0 in 2008 to 1 in 2018. Although this assessment was relative comparative, these phenomenon indicated that the express development maintain consistency to the level of socio-economic development, even play key role in promoting economic development

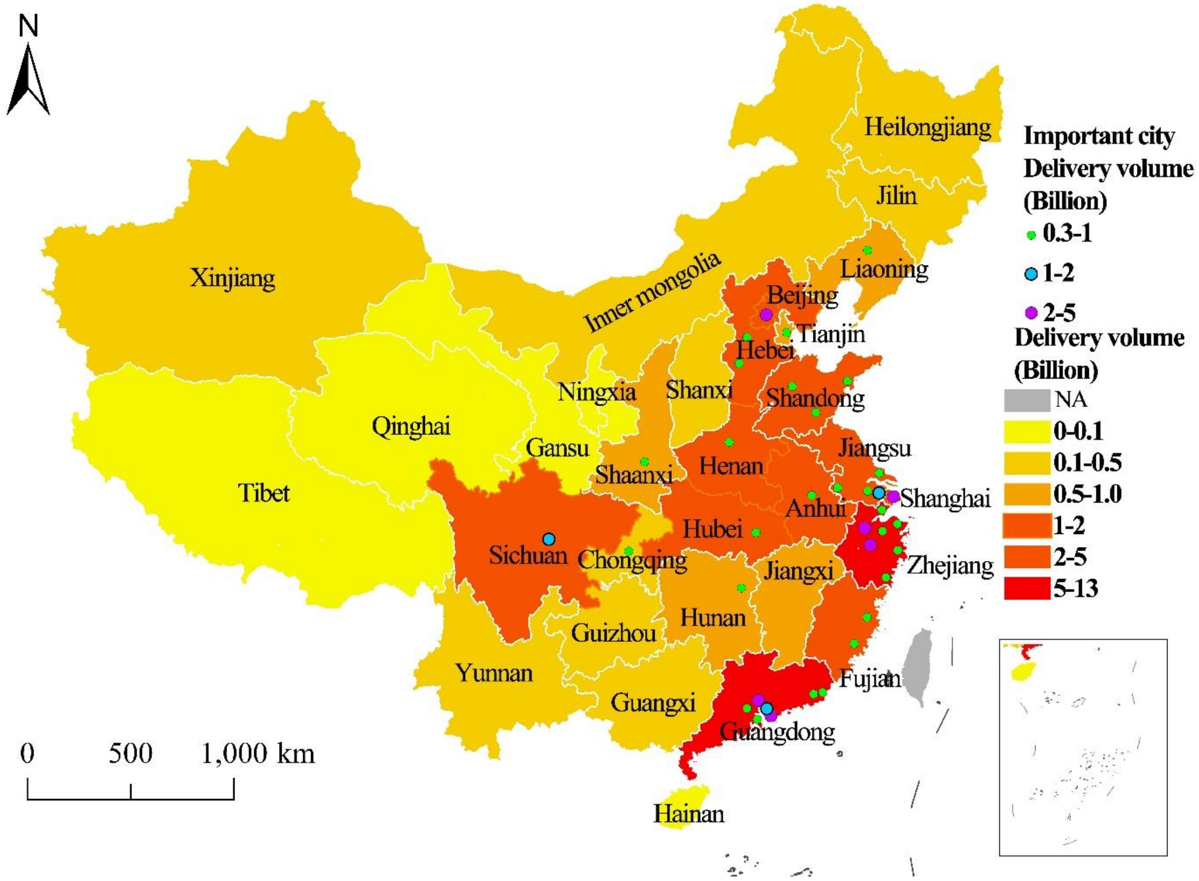

Note: the volume of intra-city and inter-city express delivery sourced from Bulletin on Postal Development Statistics of local post bureau.

Supplementary Fig.5 Quantity of express deliveries (by piece) in different provinces in mainland China (31 provinces and municipality)

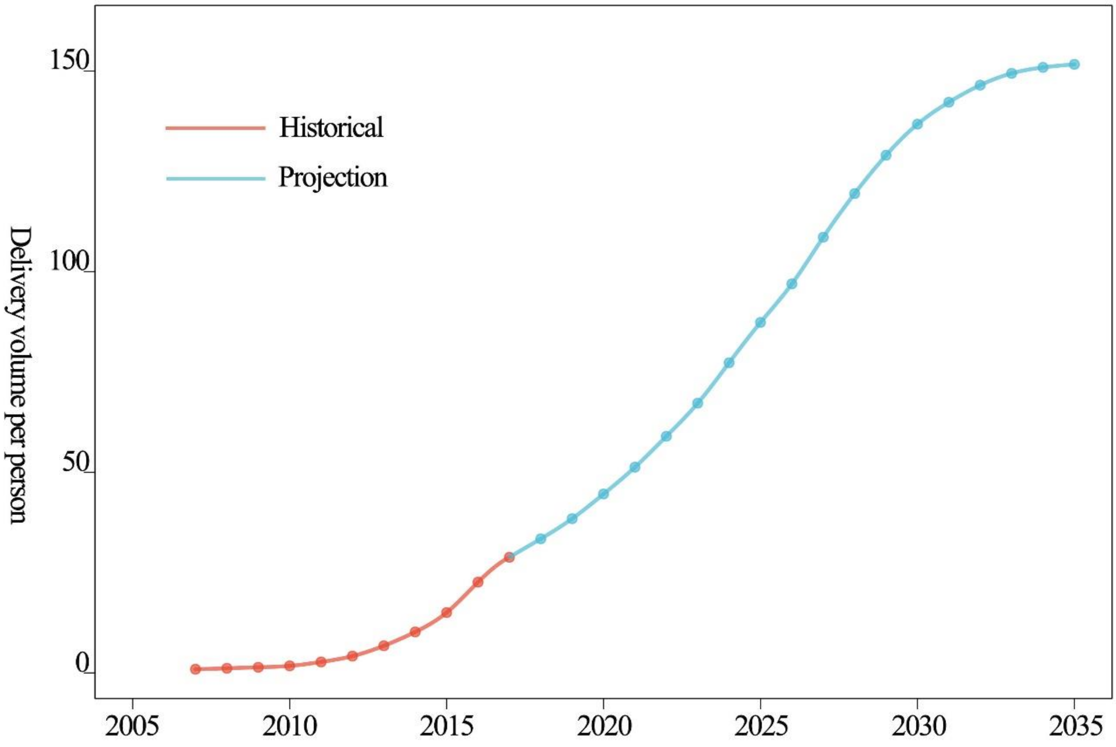

a. the projection of express received per person

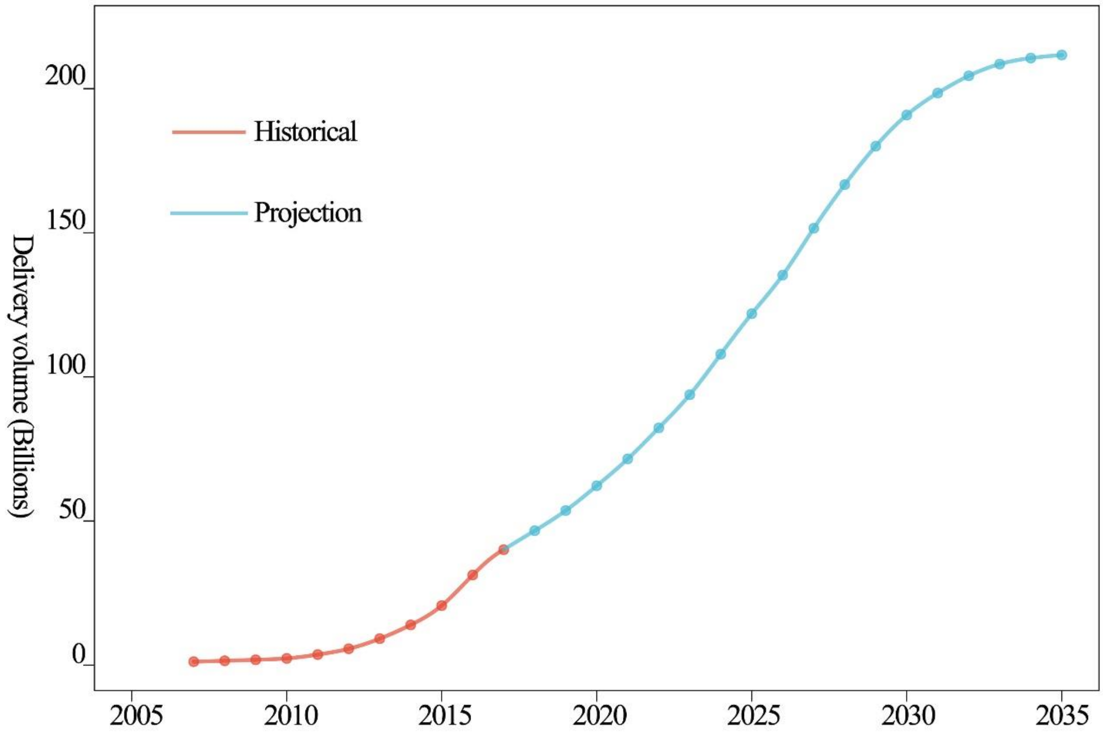

b the projection of the national delivery volume

Supplementary Fig.6 Projection of express receive per person and the express delivery volume in China

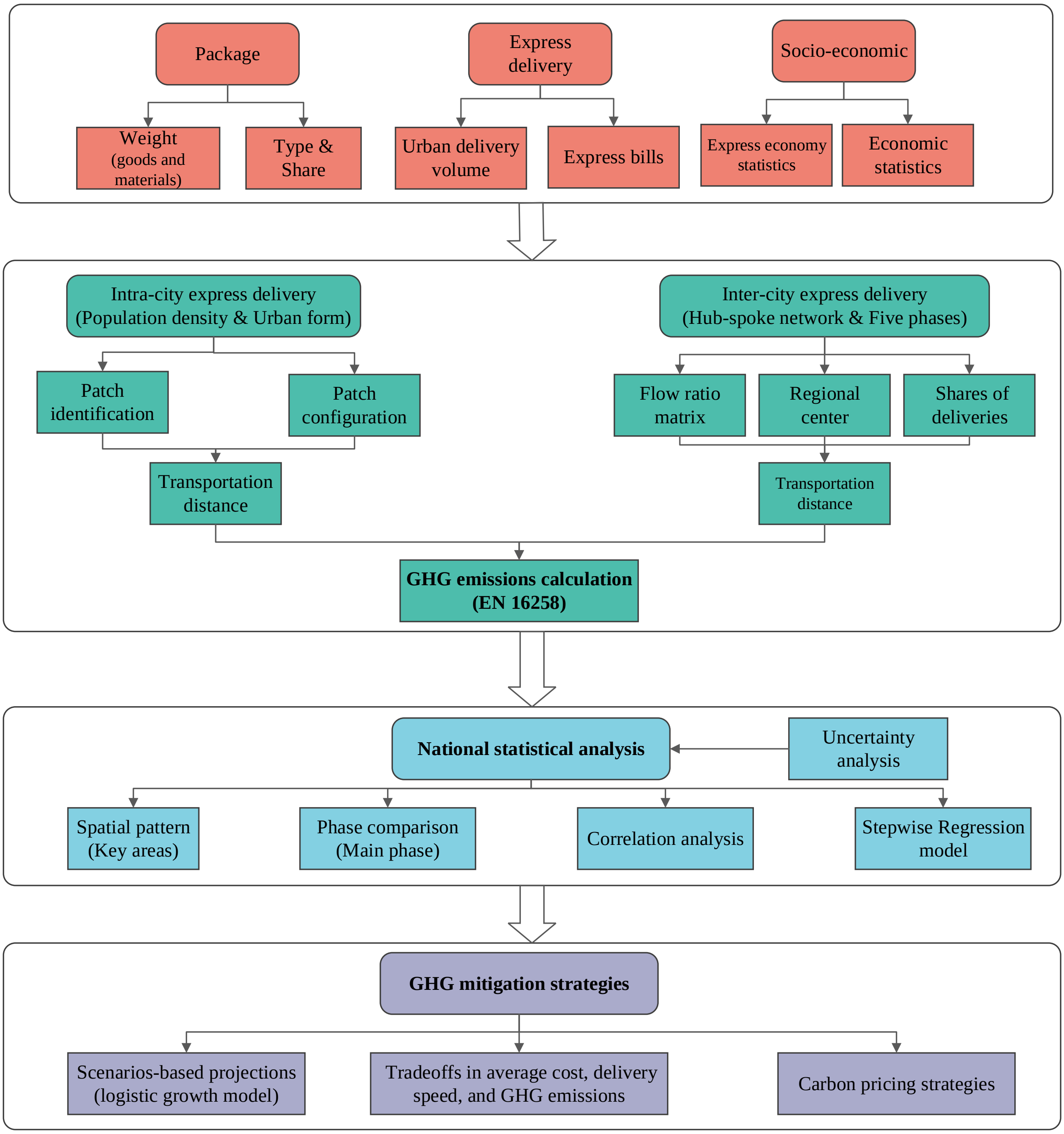

Supplementary Fig.7 Technology route of this study

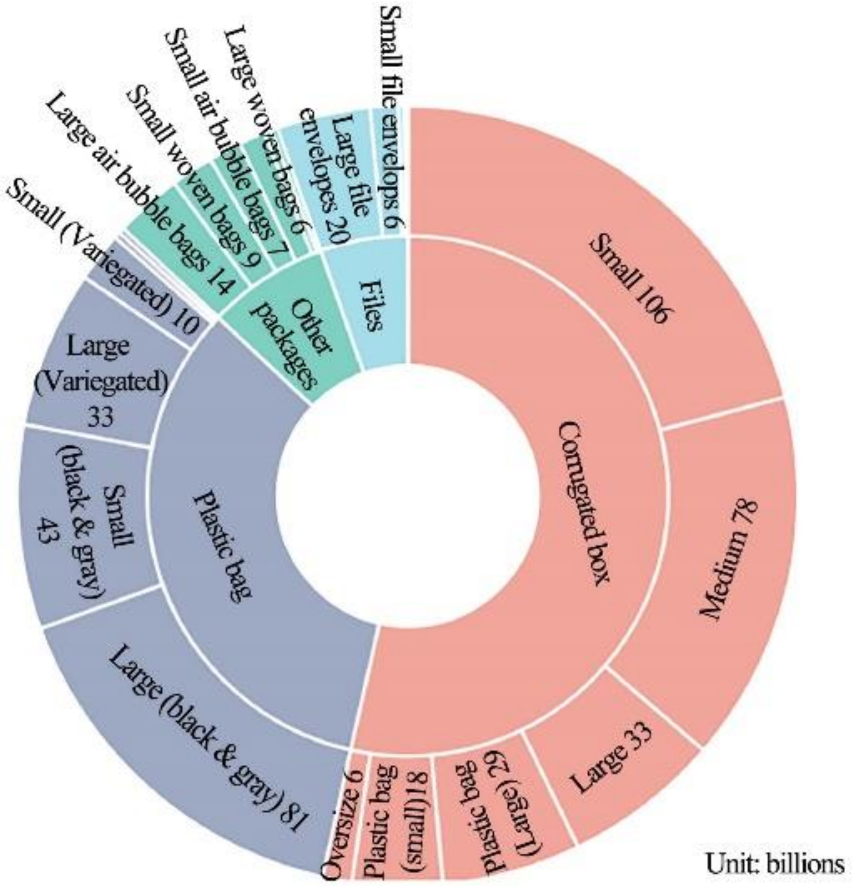

Supplementary Fig.8 Estimated composition of express deliveries (by package materials type) in China in 2018 (by piece)

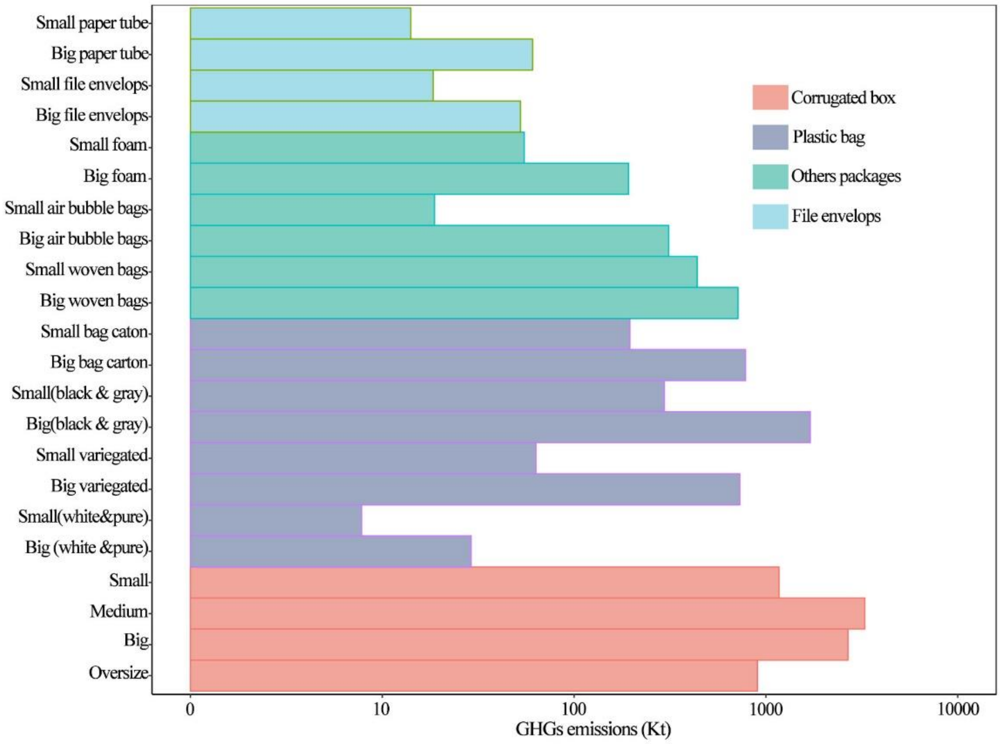

Supplementary Fig.9 Total GHG emissions estimate from the logistics and transportation of online shopping parcels: divided by types of pracles or packages materials (2018)

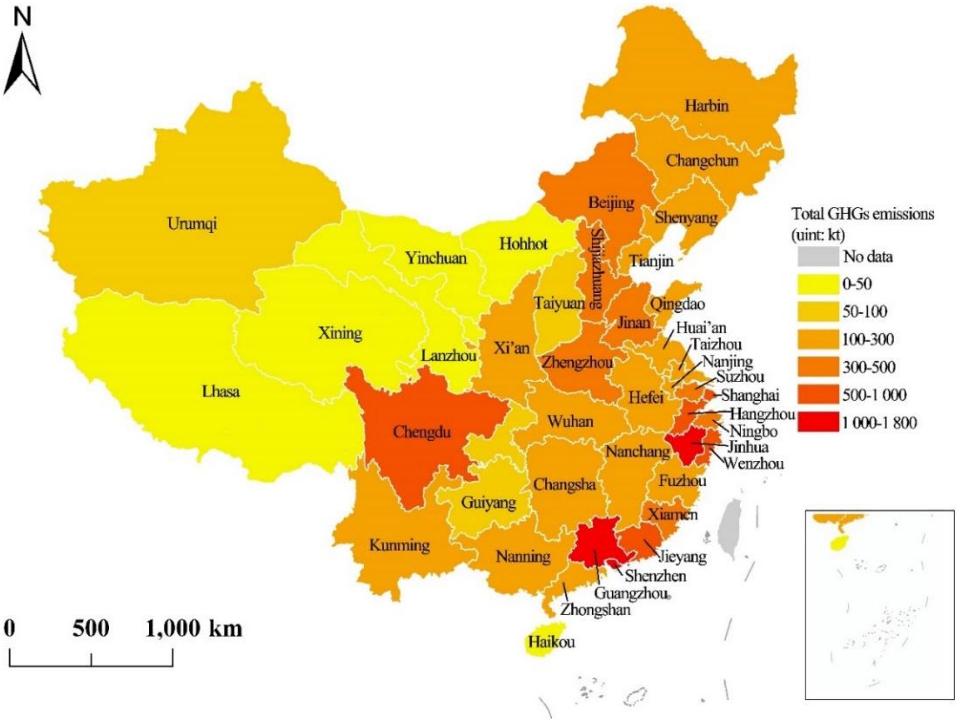

(A)

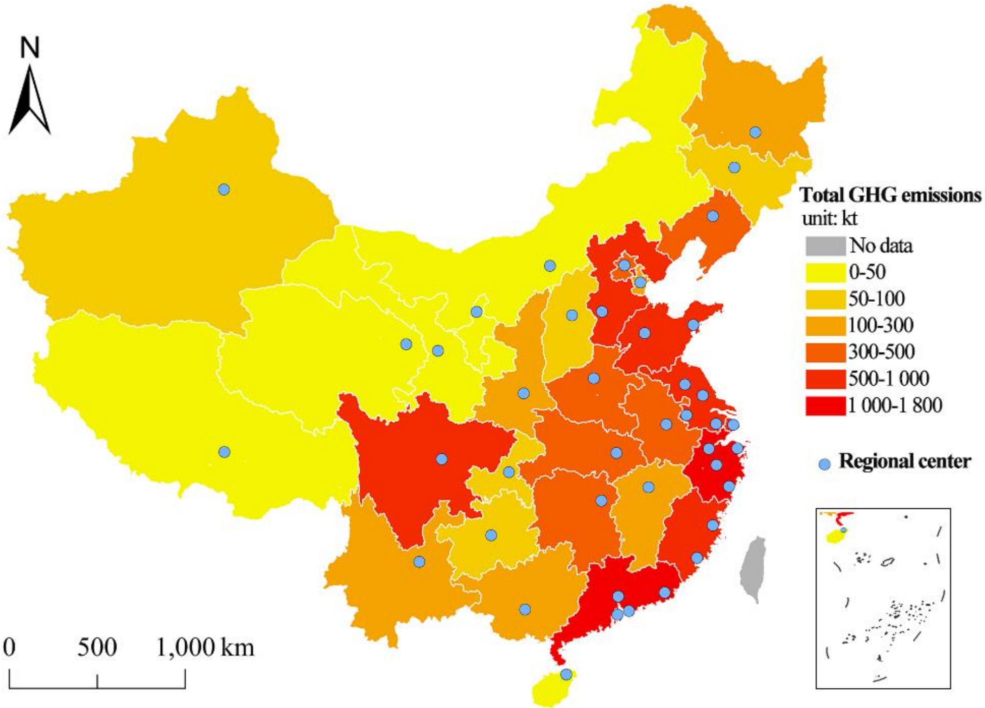

(B)

Note: total GHG emissions of regional and provinces were calculated by our model of express delivery.

Supplementary Fig.10 Total GHG emissions from the logistics and transportation of express delivery (intra-cities and inter-cities delivery): (A) by regional centers; (B) By provinces. Each region covers areas within the distribution radius from a regional center (a city) represented by a dot.

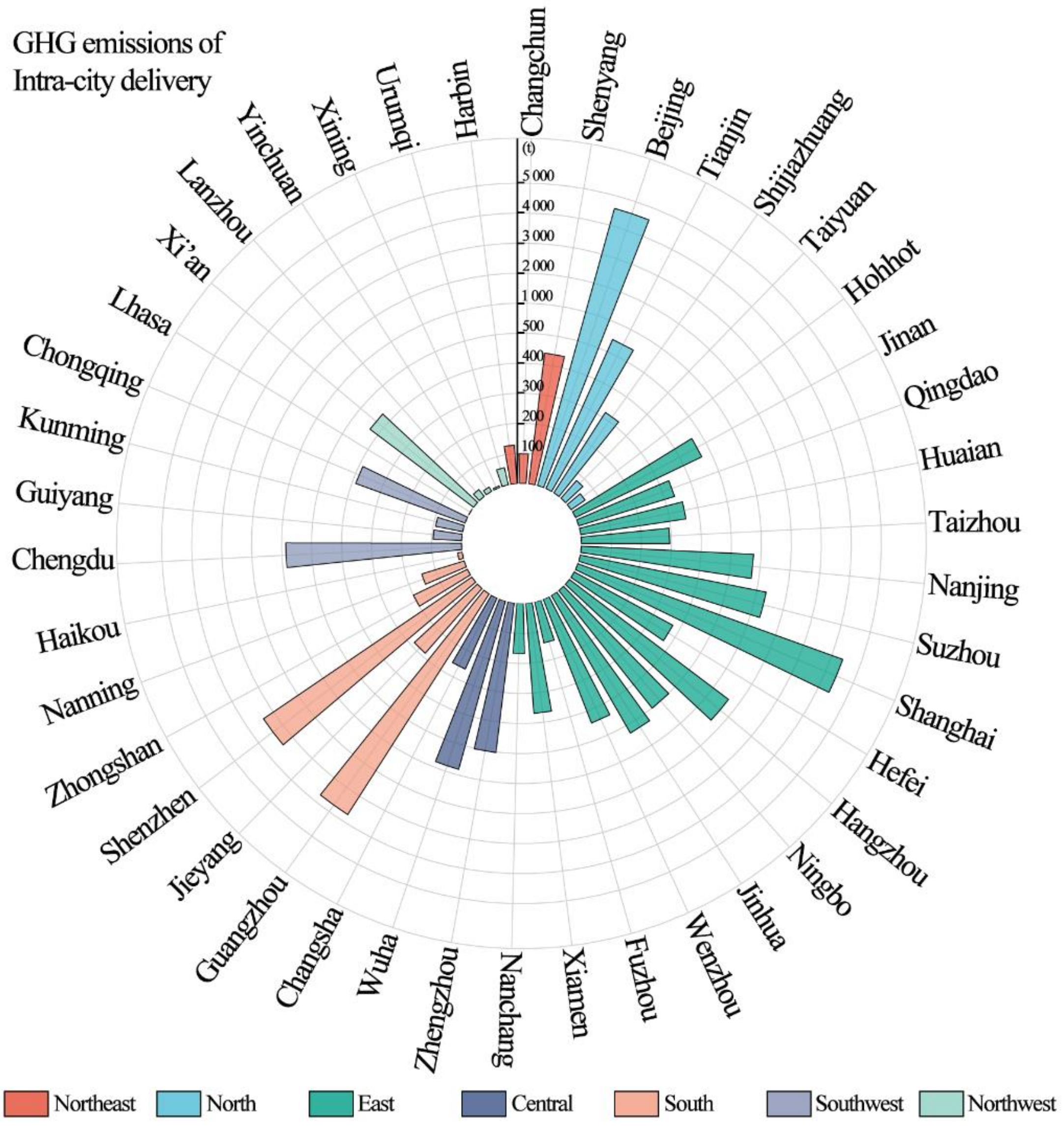

Supplementary Fig.11 Total GHG emissions from the logistics and transportation of intra-cities express delivery (by regional centers/cities)

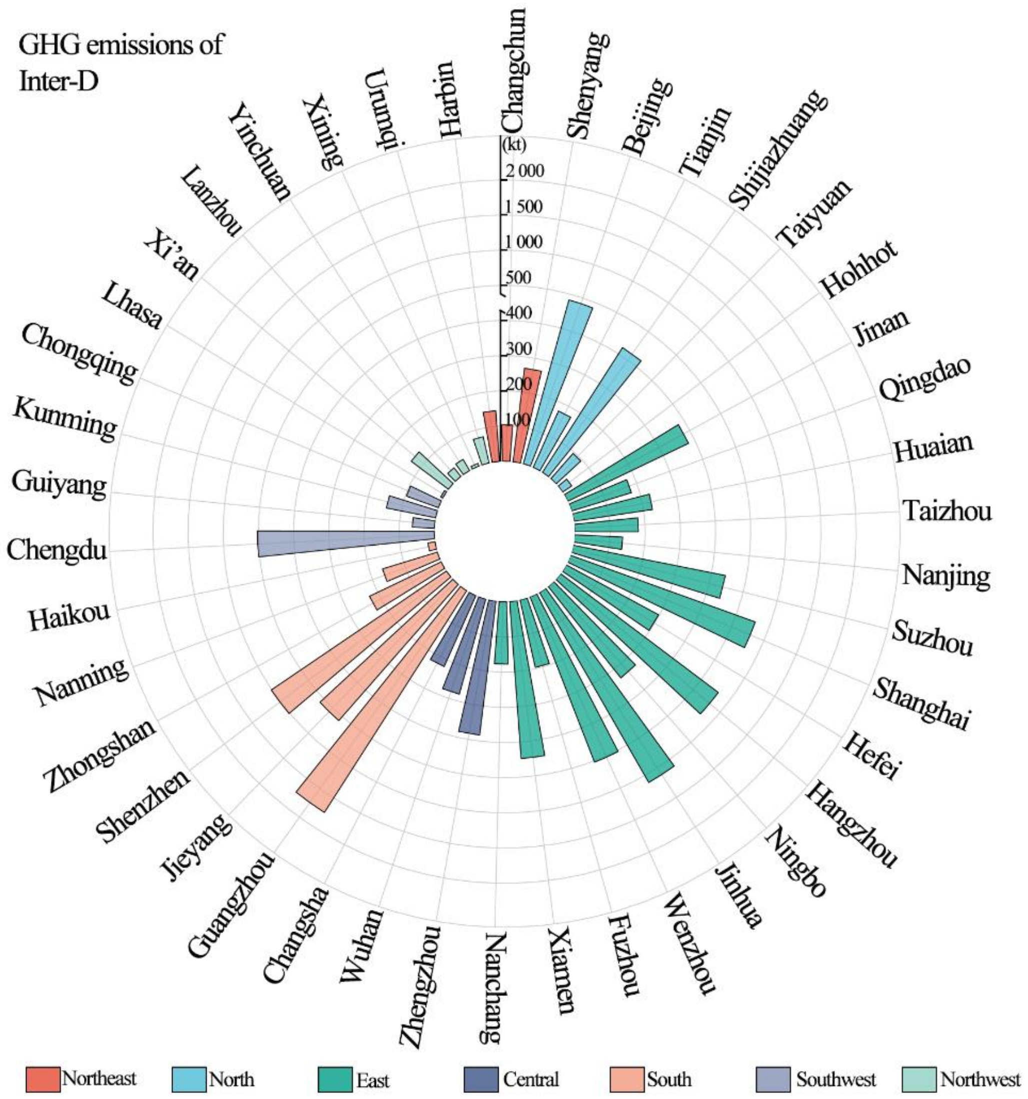

Supplementary Fig.12 Total GHG emissions from the logistics and transportation of inter-cities express delivery (by regional centers/cities)

The maximum GHG emission from the shipment of Intra-city service was mainly located in Beijing and Shanghai city, while the maximum GHG emission from the shipment of Intra-city service was mainly located in Guangzhou and Shenzhen city. The minimum GHG emissions were mainly located in Xining and Lhasa city.

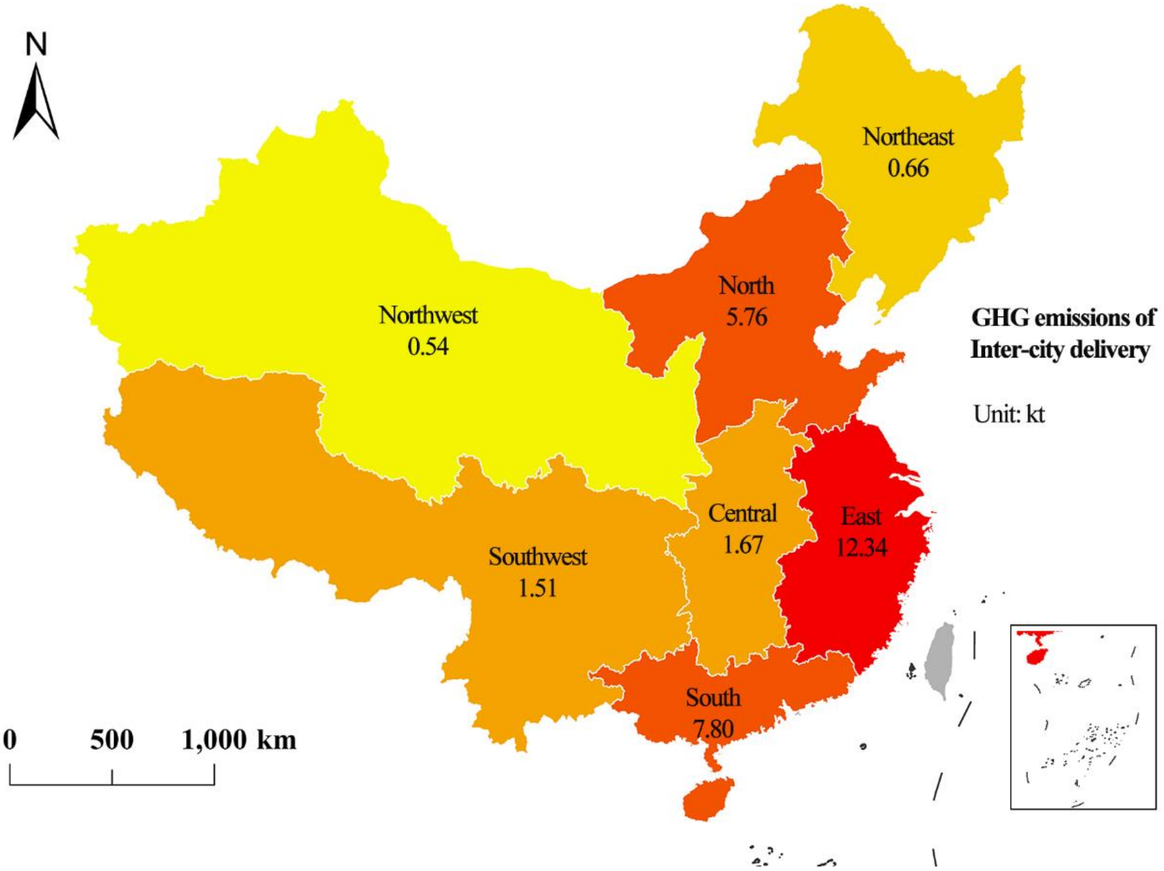

Note: GHG emissions of seven geographical regions were calculated by our model of intra-city express delivery.

Supplementary Fig. 13 GHG emissions from the logistics and transportation of intra-cities express delivery (by seven geographical regions)

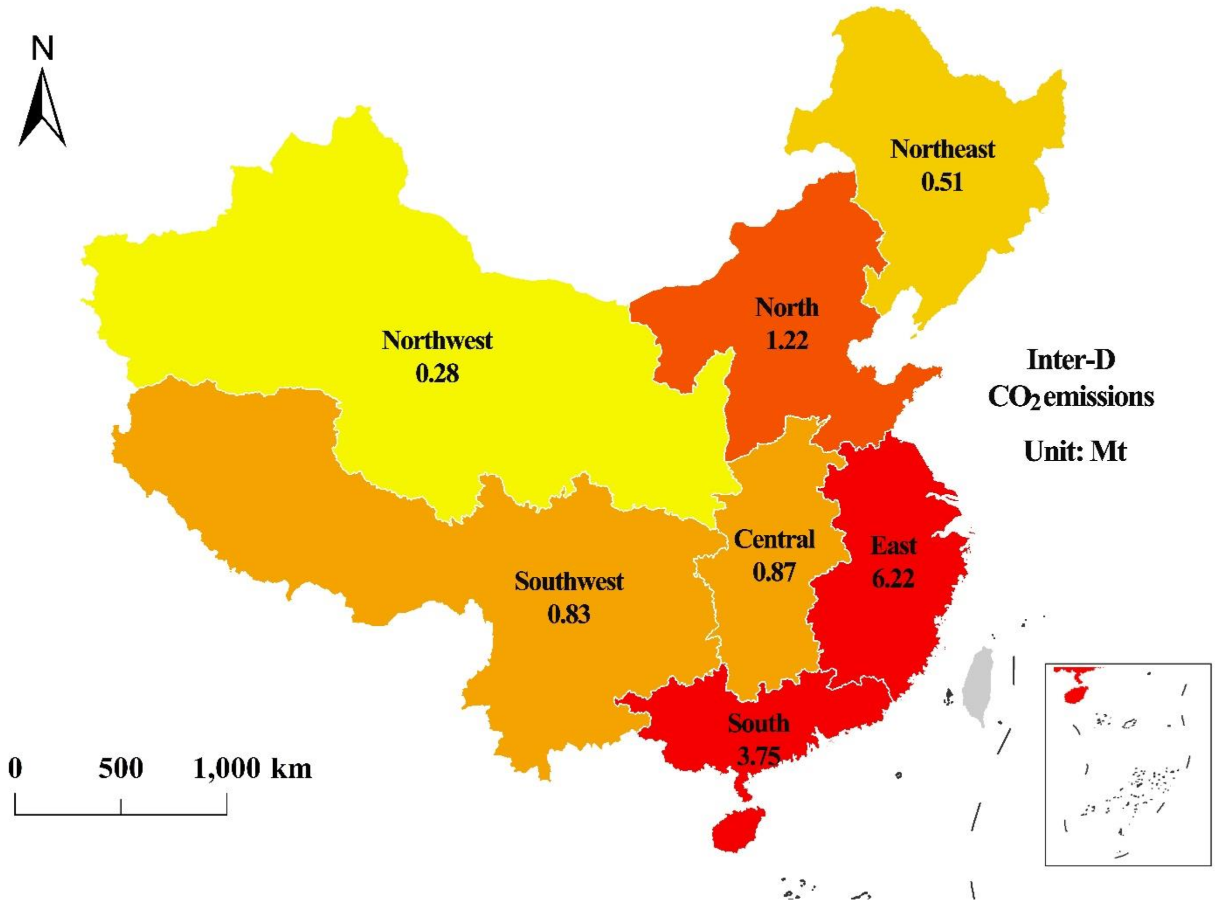

Note: GHG emissions of seven geographical regions were calculated by our model of inter-city express delivery.

Supplementary Fig.14 GHG emissions from the logistics and transportation of inter-cities express delivery (by seven geographical regions)

As can be seen in Figure S10 and S11, the GHG emission from the shipment of Inter-city express delivery was much higher the counterpart of Intra-city express delivery. The maximum GHG emission from the shipment of parcels was mainly located in the East and South of China, while the low emissionwas located in the Southwest and Northwest of China.

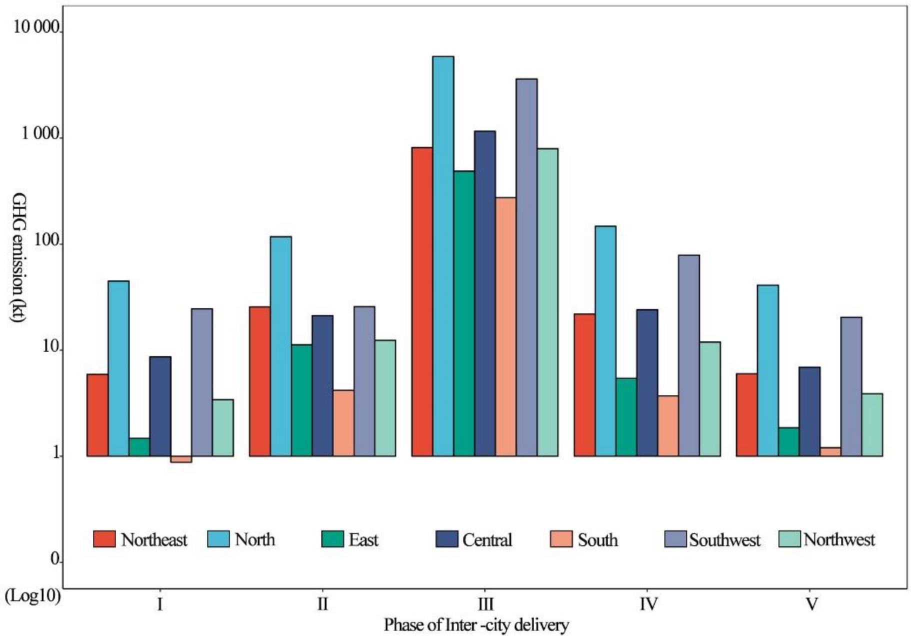

Supplementary Fig.15 GHG emissions from the logistics and transportation of express delivery (inter-city express delivery, Phase I to V)

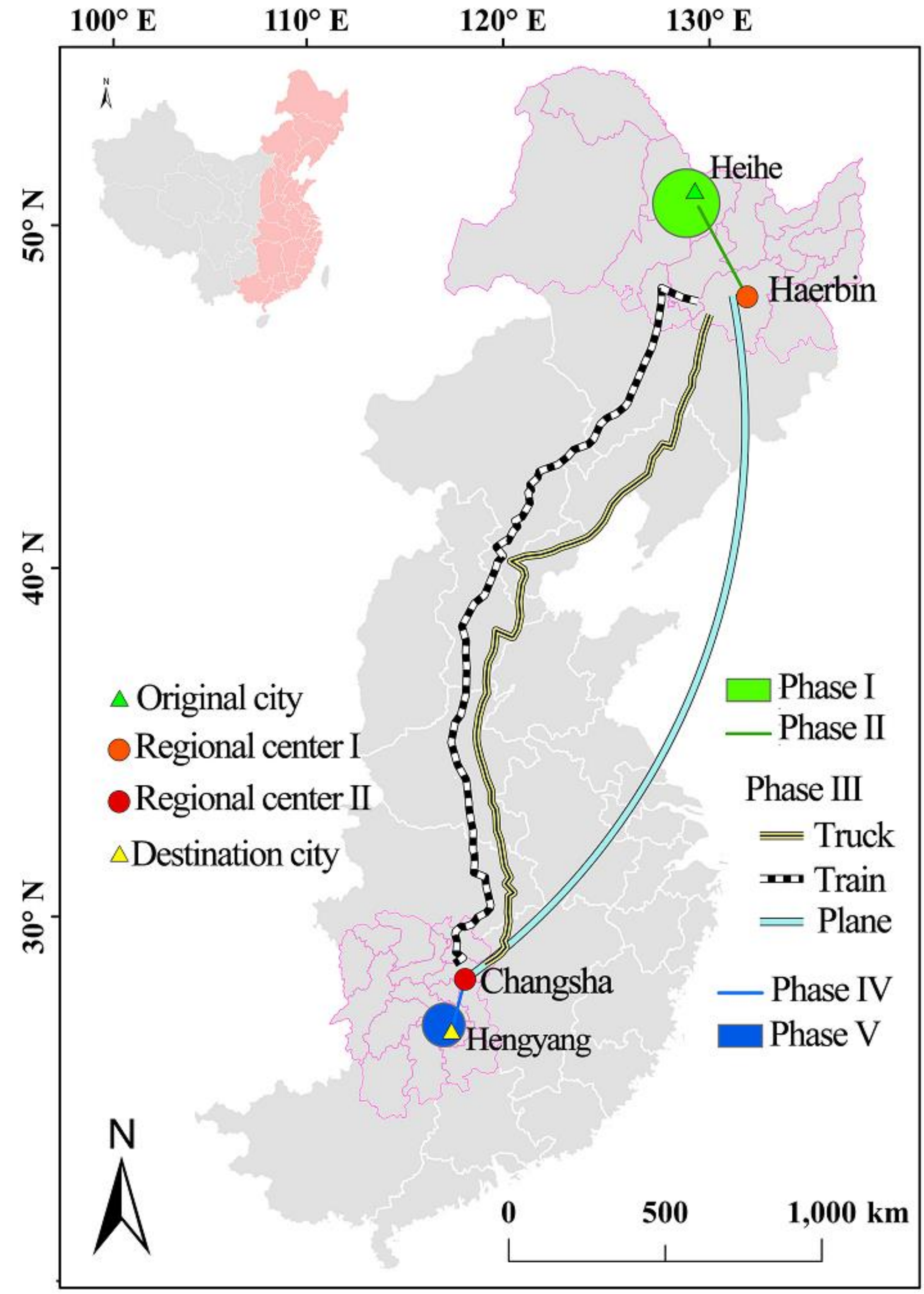

Note: there are five processes in inter-city express delivery. These processes cover four cities, including delivery city, one regional center, the other regional center, and receiving city. I) the inter-city express delivery in delivery city 'Heihe'; II) the branch line transportation (from the distribution center of Heihe to the regional center 'Harbin'; III) the mainline transportation from the regional center 'Harbin' to another regional center 'Changsha'; IV) the branch line transportation from regional center 'Changsha' to the receiving city 'Hengyang'; V)the inter-city express delivery in receiving city 'Hengyang'.

Supplementary Fig.16 Five transport phases of inter-city express delivery: a case study

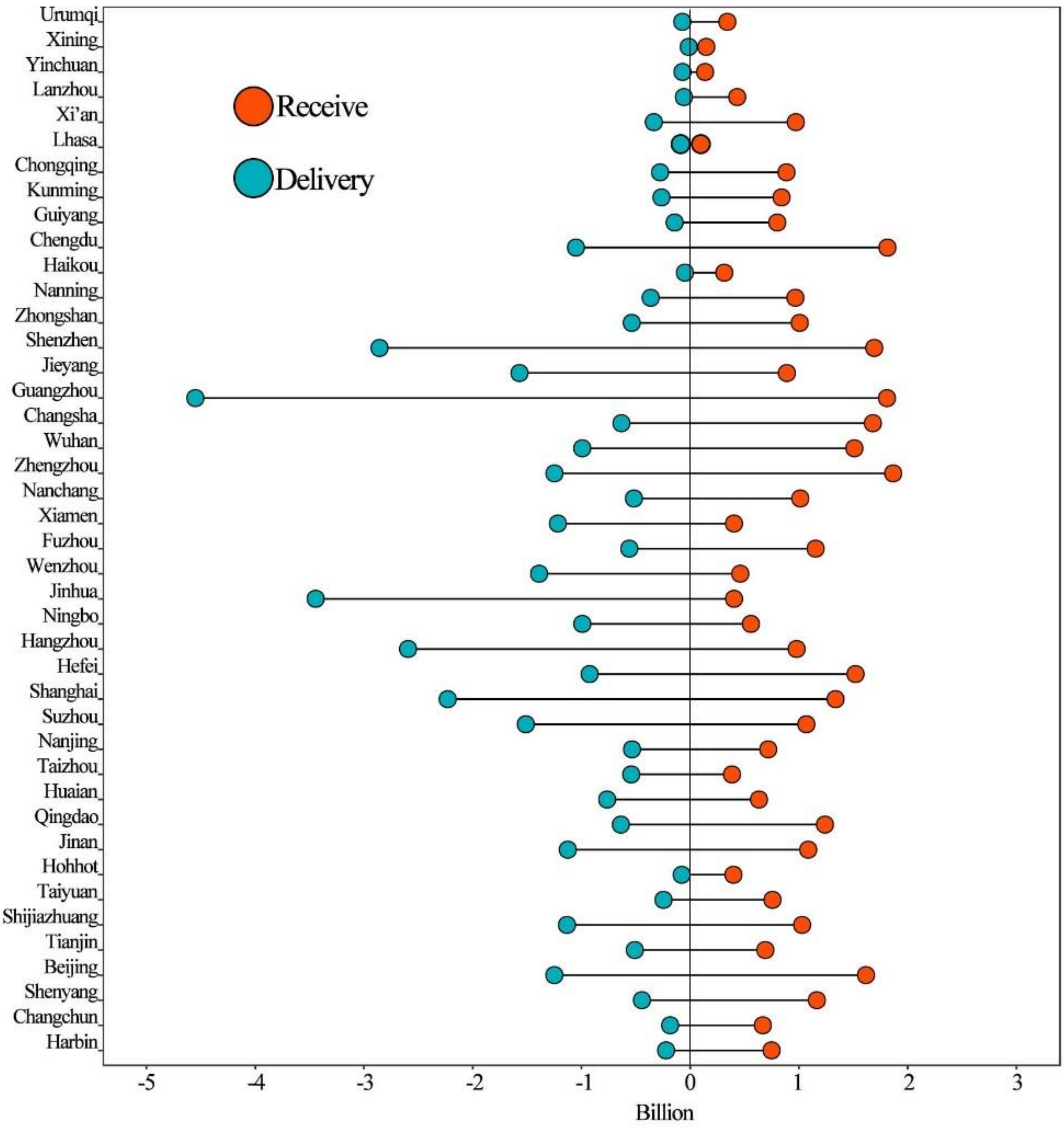

Supplementary Fig.17 Delivery volume and receive volume of express deliveries for each regional center

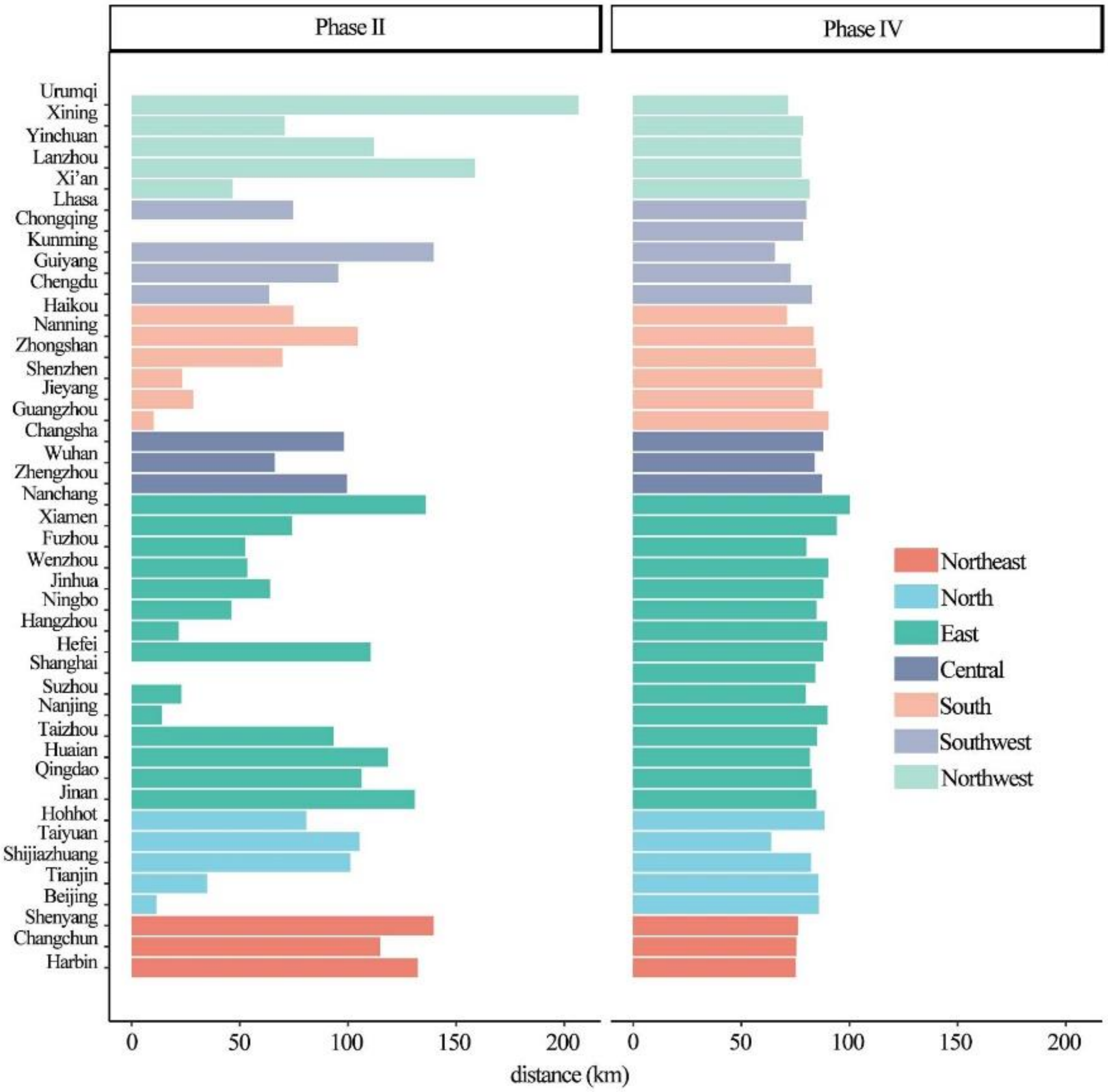

Supplementary Fig.18 Average distance of phase II and IV of inter-city express delivery

Conversely, the transportation phase II or IV of inter-city express was the road distance from a single city to the regional center. The range of mean distance of the regional center was from 0 to 650km. The regional center of Shanghai and Chongqing don't own phase II and V. Obviously, the distance of regional center in northeast and northwest were longer than another regional center. However, the distance of regional center in east and south was lower than 150km.

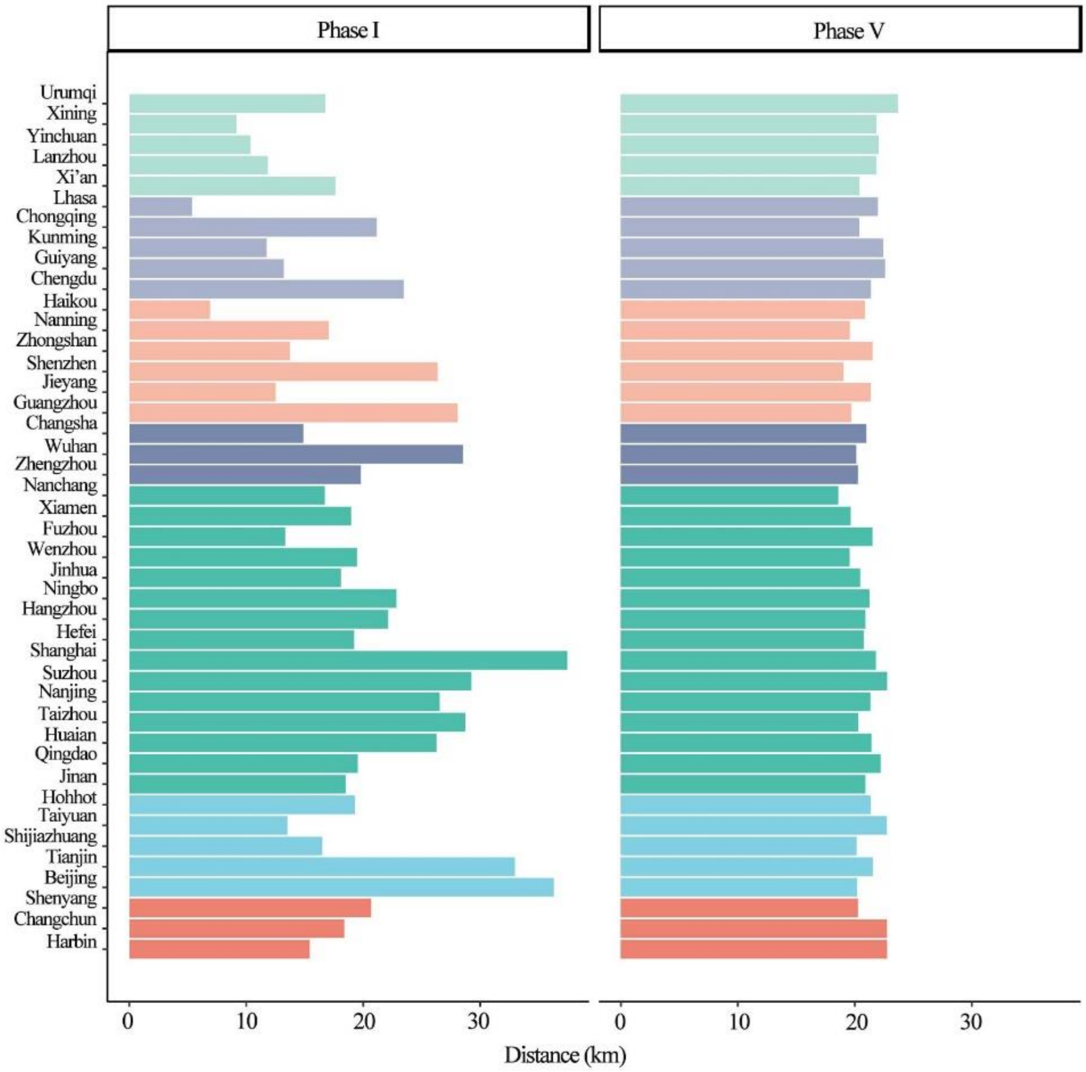

Supplementary Fig. 19 Transport distance of phase II and IV (average value) of inter-city express delivery

The range of mean distance was from 5km to 37km in phase I. Thereof, the mean distance of the regional center in eastern China was longer than another regional center, while the distance of the regional center in Northeast and Northwest were lower. That was attributed to that built-up area and population density in the eastern coastal province were more than this counterpart of the western province. The reason for the GHG emission of the phase I was more than phase V was that the mean distance of intracity express in the eastern regional center was longer than the counterpart of the western regional center.

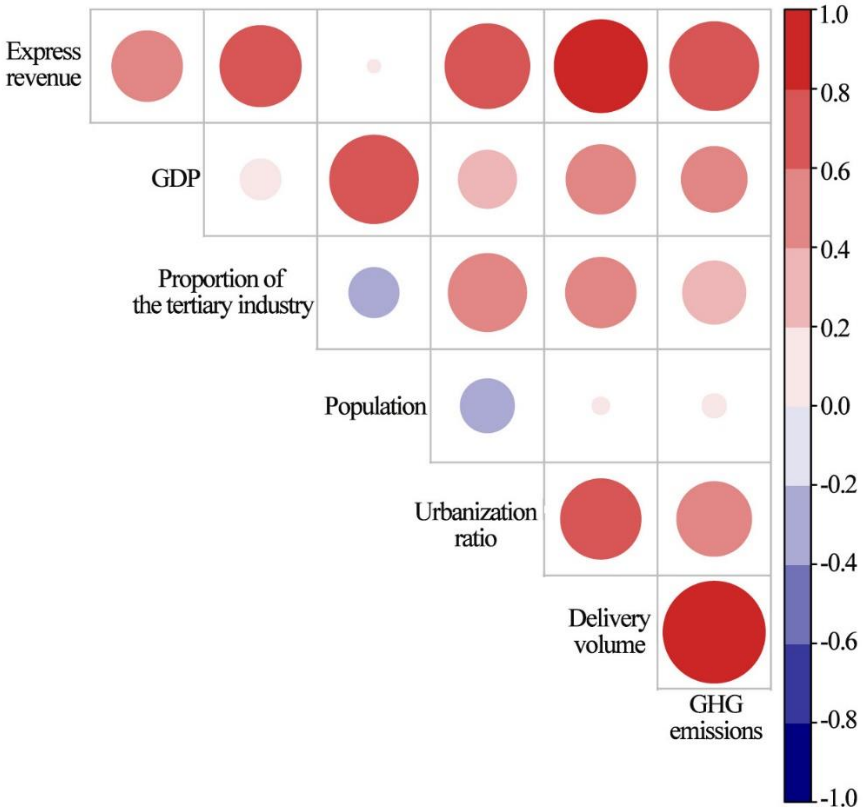

Supplementary Fig.20 Correlation ship analysis between social-economic factors and GHG emissions from the shipment of express delivery of inter-city express delivery (correlation analysis from the total volume).

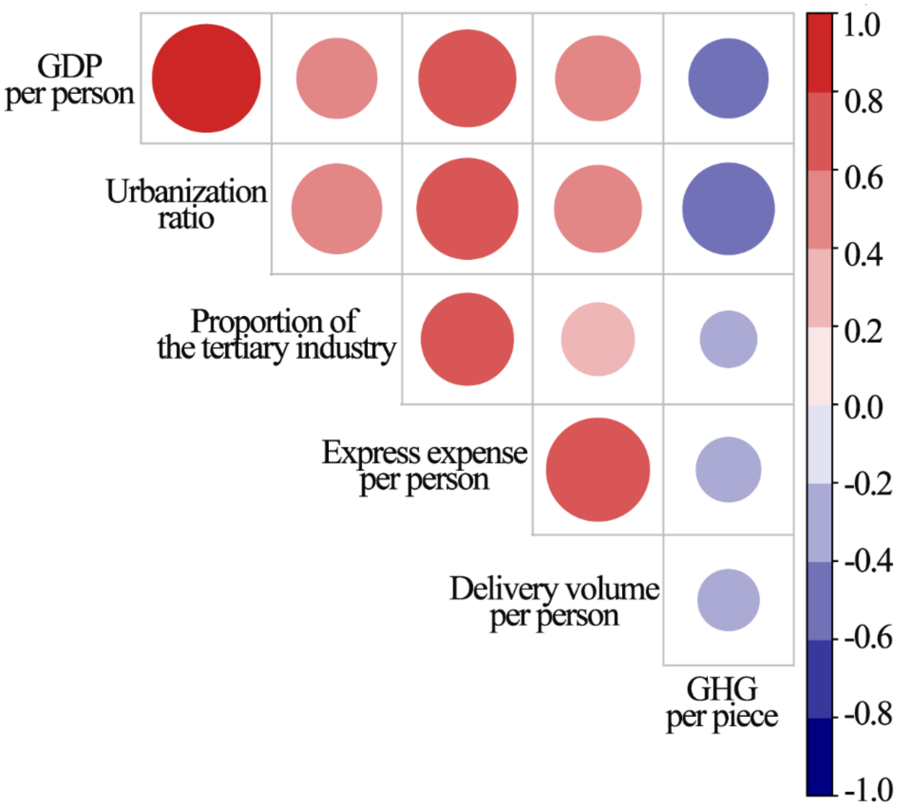

Supplementary Fig.21 Correlation ship analysis between social-economic factors and GHG emissions from the shipment of express delivery of inter-city express delivery (correlation analysis from per piece).

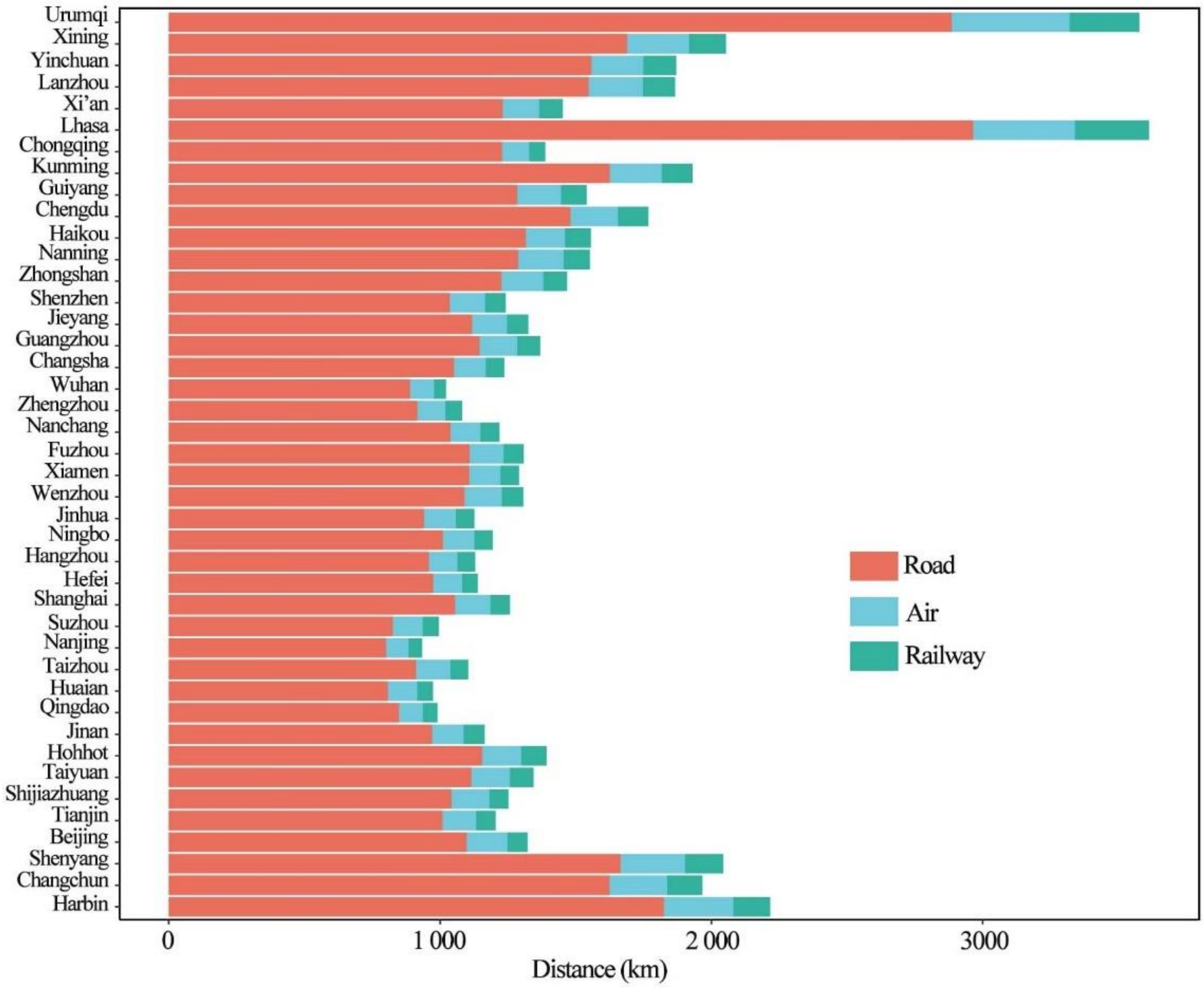

Supplementary Fig.22 Distance of three types of transport type for each piece of parcel of inter-city express delivery

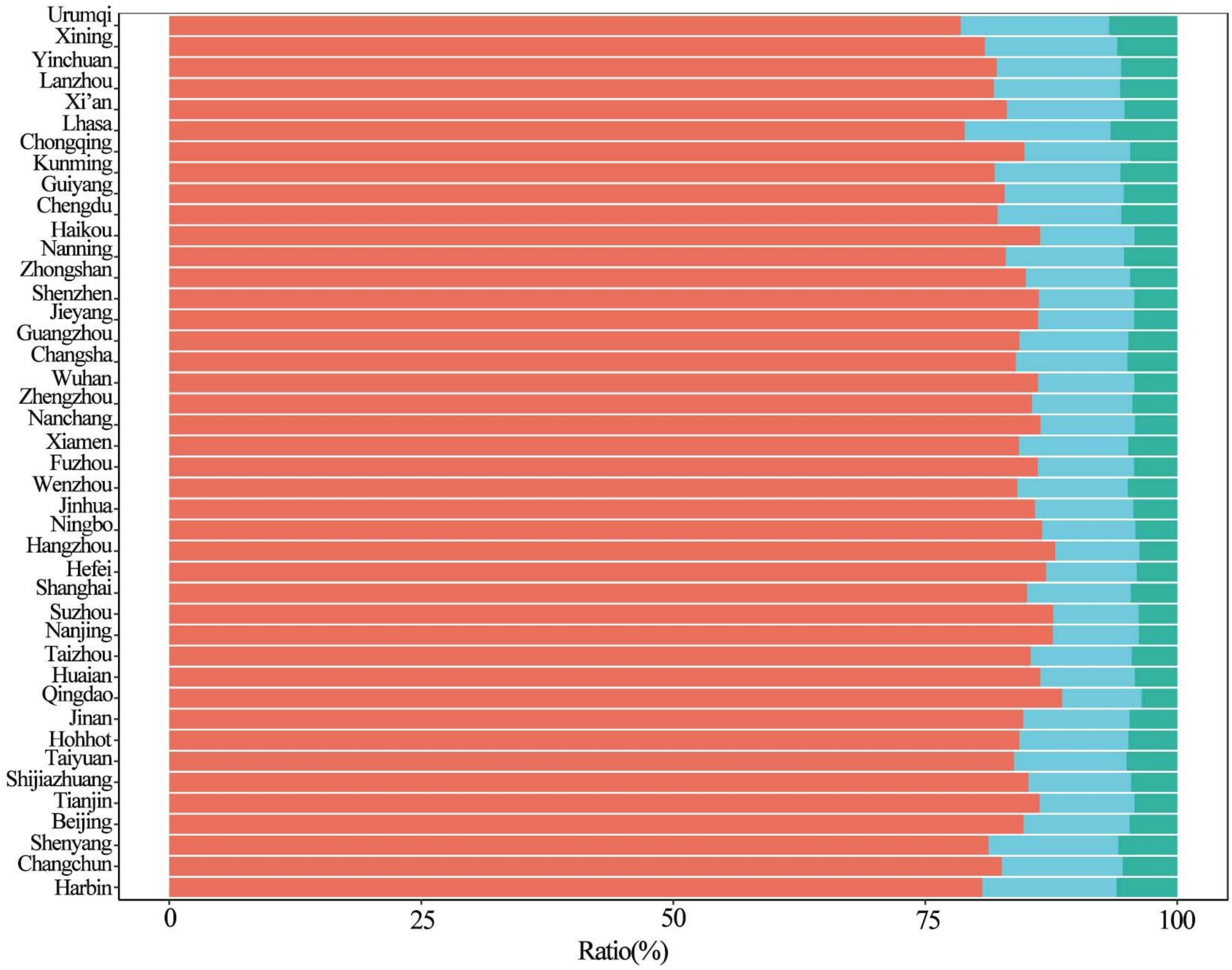

Supplementary Fig.23 Proportion of three types of transportation mode for each piece of parcel of inter-city express delivery

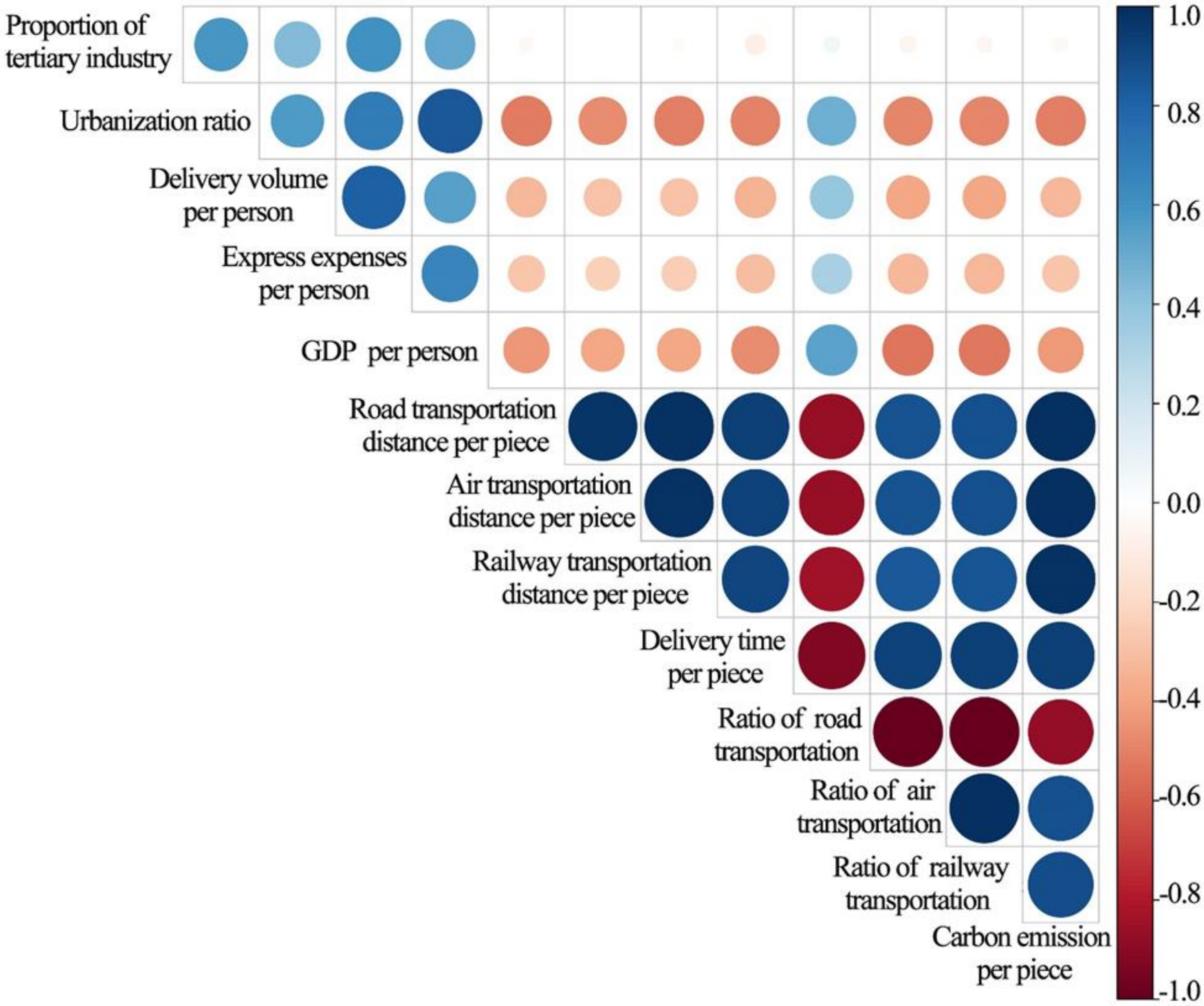

Supplementary Fig. 24 Correlation ship analysis between social-economic factors and GHG emissions from the shipment of express delivery of inter-city express delivery

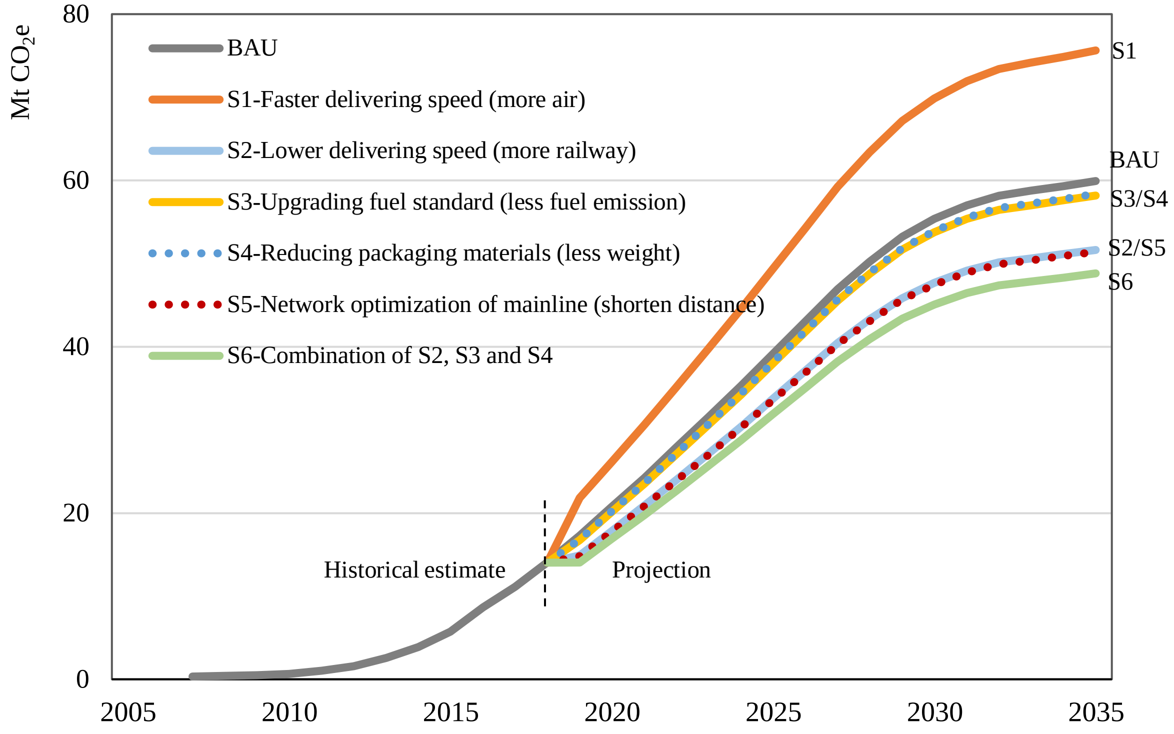

Supplementary Fig.25 GHG emissions from express deliveries under various scenarios in China. A nearly overlap between S3 and S4, S2 and S5.

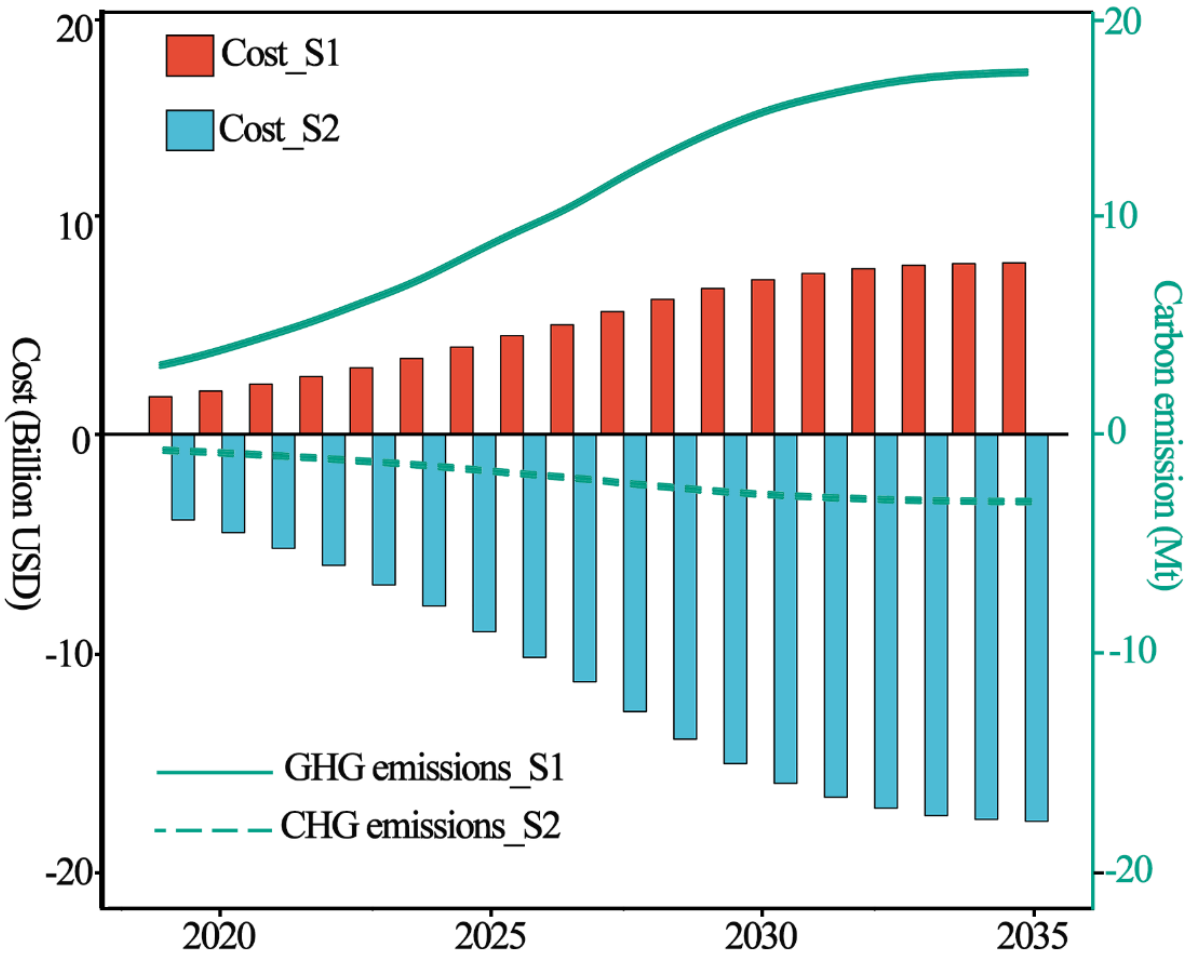

Supplementary Fig. 26 An estimate of the GHG emissions mitigation potentials subject the adjustment of delivery mode for inter-city express delivery service: Tradeoff between Speed (cost) and carbon saving

Faster delivery (S1) uses vehicles with higher GHG emissions, but requires less delivery times, which is paid more fees by consumers. Conversely, slower delivery (S2) uses vehicles with lower GHG emissions, but requires longer delivery times, which is paid less fees by consumers.

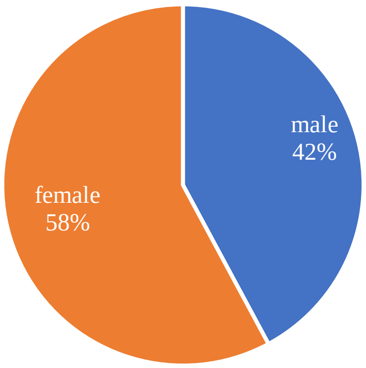

a.Sex ratio

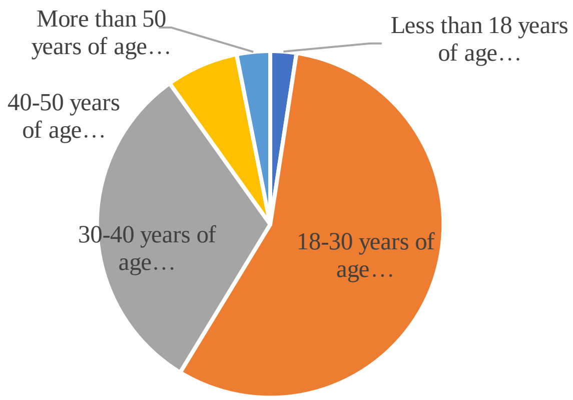

b. Age composition

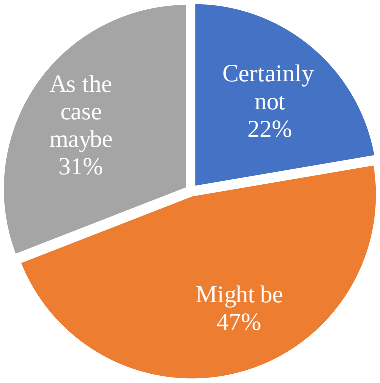

c. Research on green consumption willing of express based on carbon label

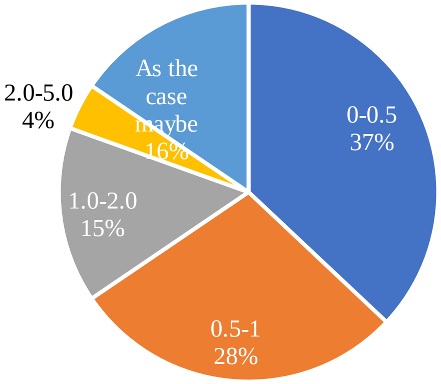

d.Willing to pay carbon taxes(CNY)

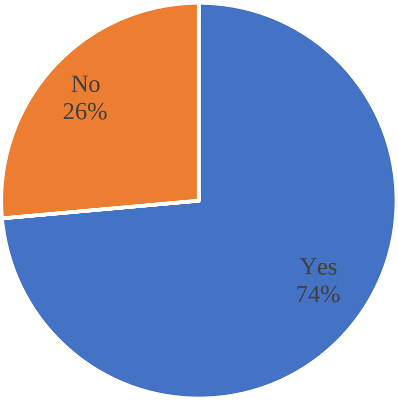

e. Willing to choose the express packaging of green degradable materials and pay a certain fee

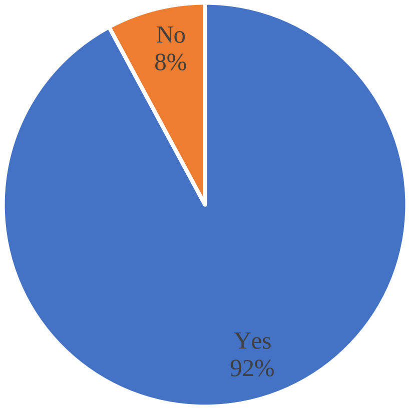

f. Would you like to cooperate with the recycling and reuse of express garbage

Supplementary Fig.27 Results of questionnaire on carbon labelling of inter-city express delivery

We used the principle of stratified sampling to distribute the formal questionnaires across China according to the distribution percentages of express delivery volume. The questionnaires

were distributed online and offline across China. Offline questionnaires were survey in the form of paper on campuses, and in communities, and online questionnaires were circulated on social media in the form of link. In this study, 1210 samples were collected with a 97.5% response rate to maintain the required responses for the survey. These attributes suggested that the respondents might have answered the questions blindly and casually, which would reduce the reliability of the questionnaires.

Therefore, potentially effective strategy to mitigate GHGs emissions from the logistic and transportation of express deliveries is carbon pricing through carbon tax, emission fees, or other similar policy mechanisms. As a benchmark, the five year average price of GHGs emissions in China's carbon trading market from 2014 to 2018 [23] adds only less than 0.1% of to the cost of parcel delivery in China (Supplementary Table 3). Based on a survey we conducted with 1,895 valid responses from all 42 regional delivery centers in China, we found that consumers have a significant willingness to pay for GHGs emissions of express deliveries by as much as 10 times the five-year average price in the carbon trading market (Supplementary Fig.26).

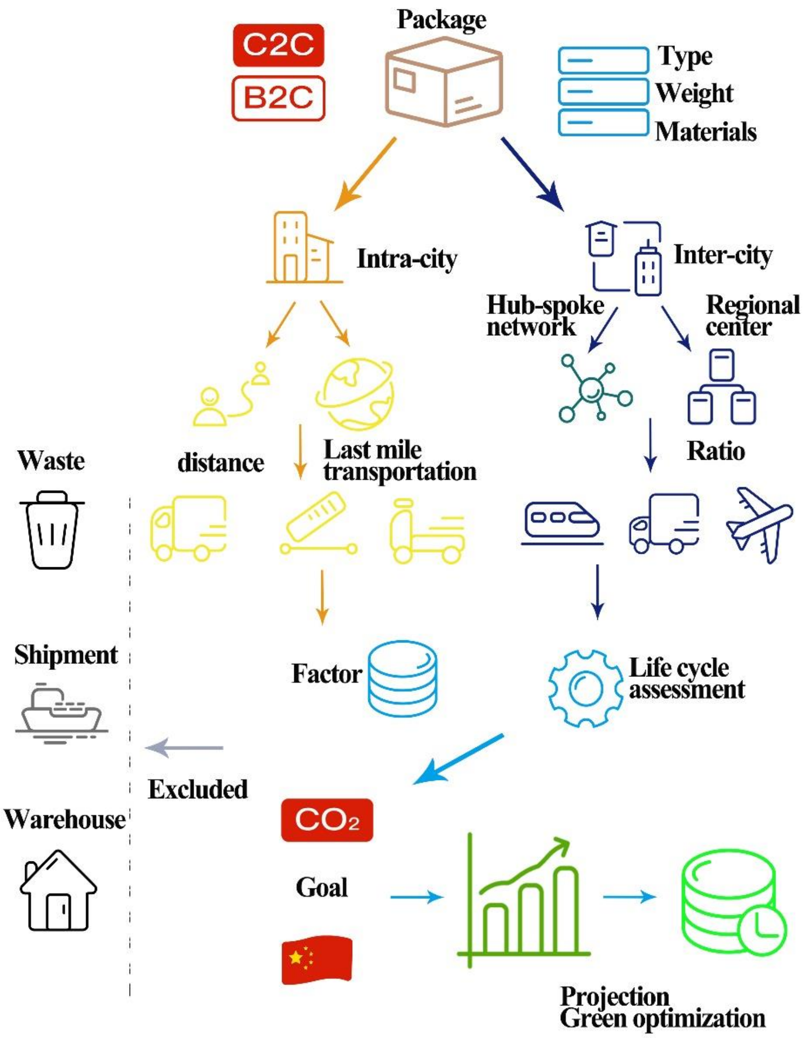

Note: our study define express delivery as the delivery service of both intra-city and inter-city parcels. Moreover, we only focus on GHG emissions, measured by CO2-equivalents from the transportation phase of domestic express deliveries, since transportation is the dominating stage of energy consumption and GHG emissions for delivery services. Here we carefully quantify the increasing environmental impacts of express delivery services and evaluate their mitigation potentials in China. These icons have been created and edited in Adobe illustrator.

Supplementary Fig.28 Goal and systemic boundary definition of this study

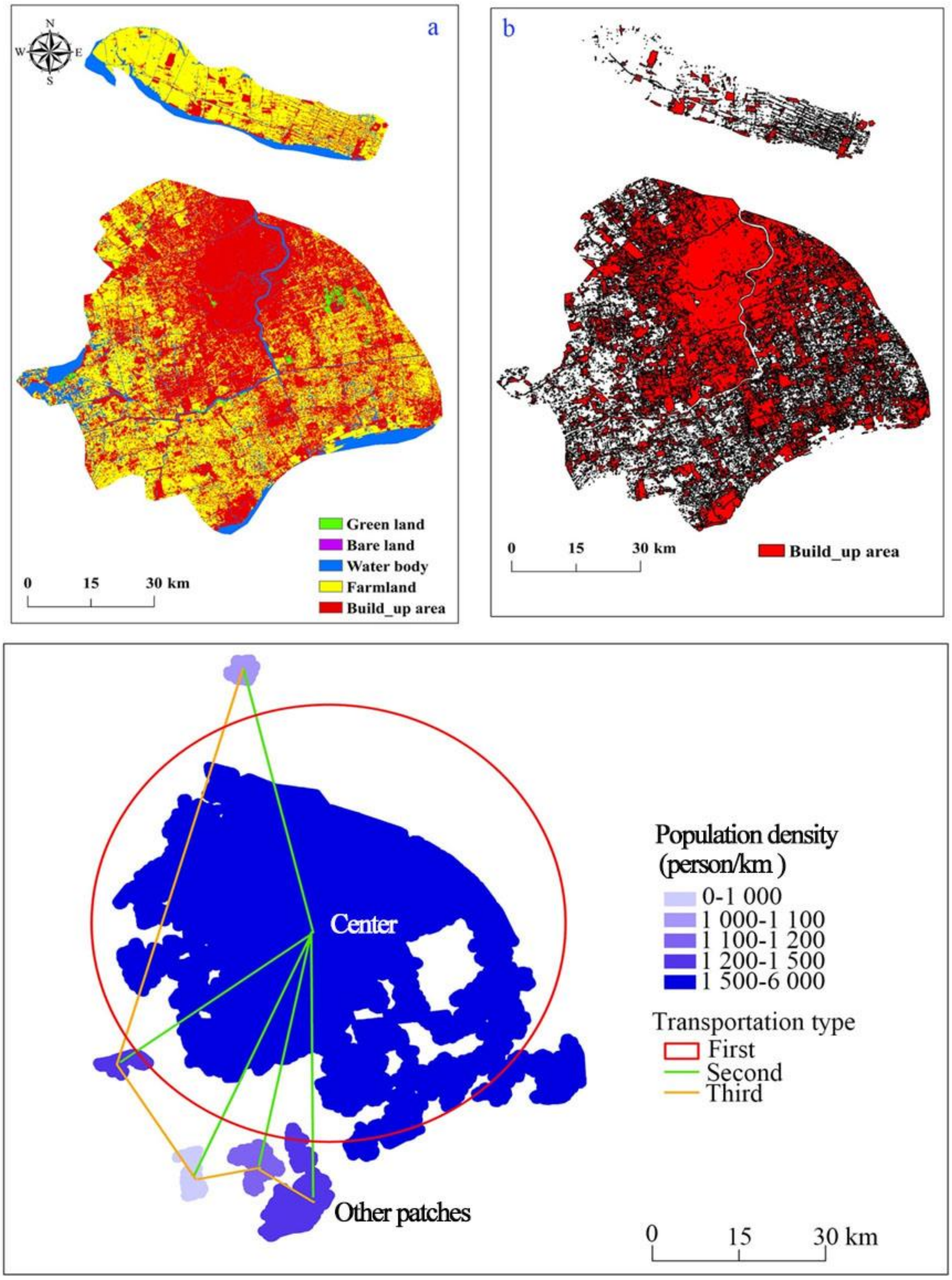

Note: there existed seven types of land cover, including green land, bare land, water body, farmland, and built-up land. Thereof, the buildup area indicated the human activity area of city. The distance for intra-city express delivery includes three parts: (1) the distance across each patch (the buildup area of human activities in the urban environment); (2) the distance between the center (the core area of human activities in the urban environment) and the other patches; and (3) the distances between the other patches.

Supplementary Fig.29 Modeling for three types of transportation phase for intra-city express delivery service- a case study of Shanghai city: (a) land cover and land use of Shanghai city; (b) buildup area of Shanghai city; (c) three types of transport phase and corresponding estimated distances.

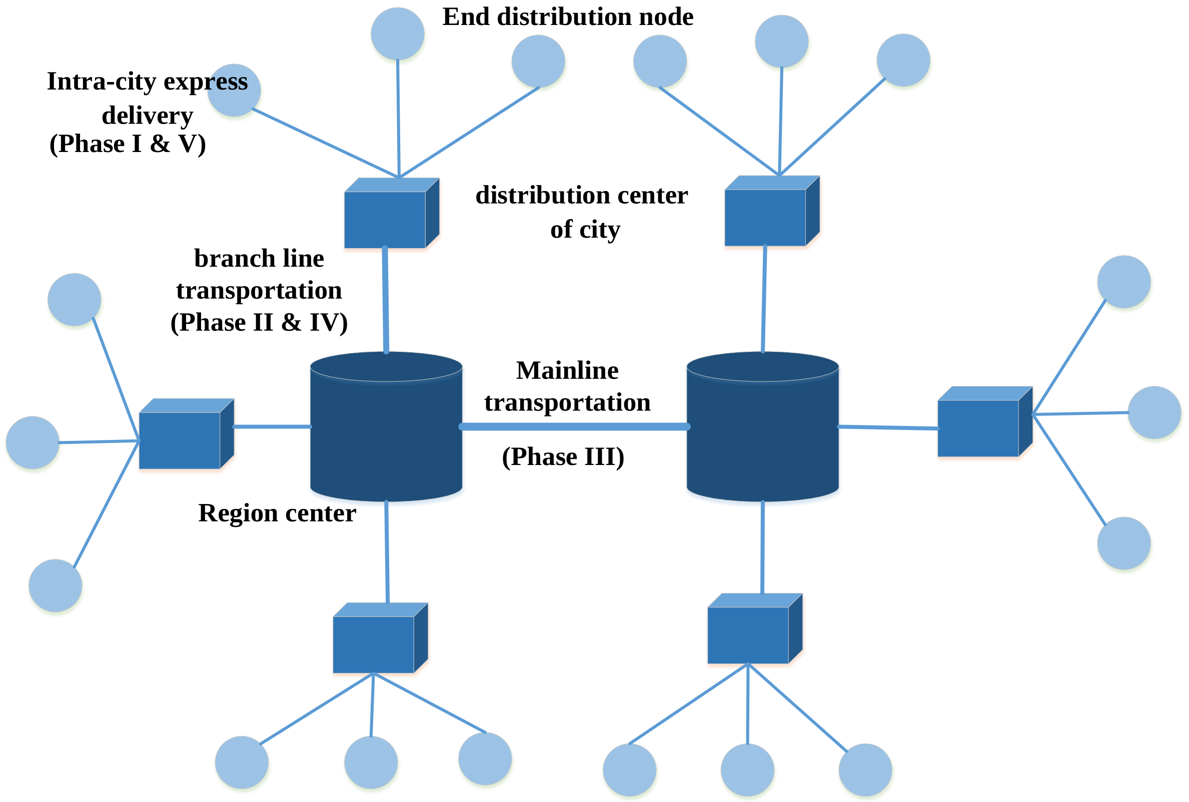

Supplementary Fig.30 The hub and spoke network of inter-city express delivery

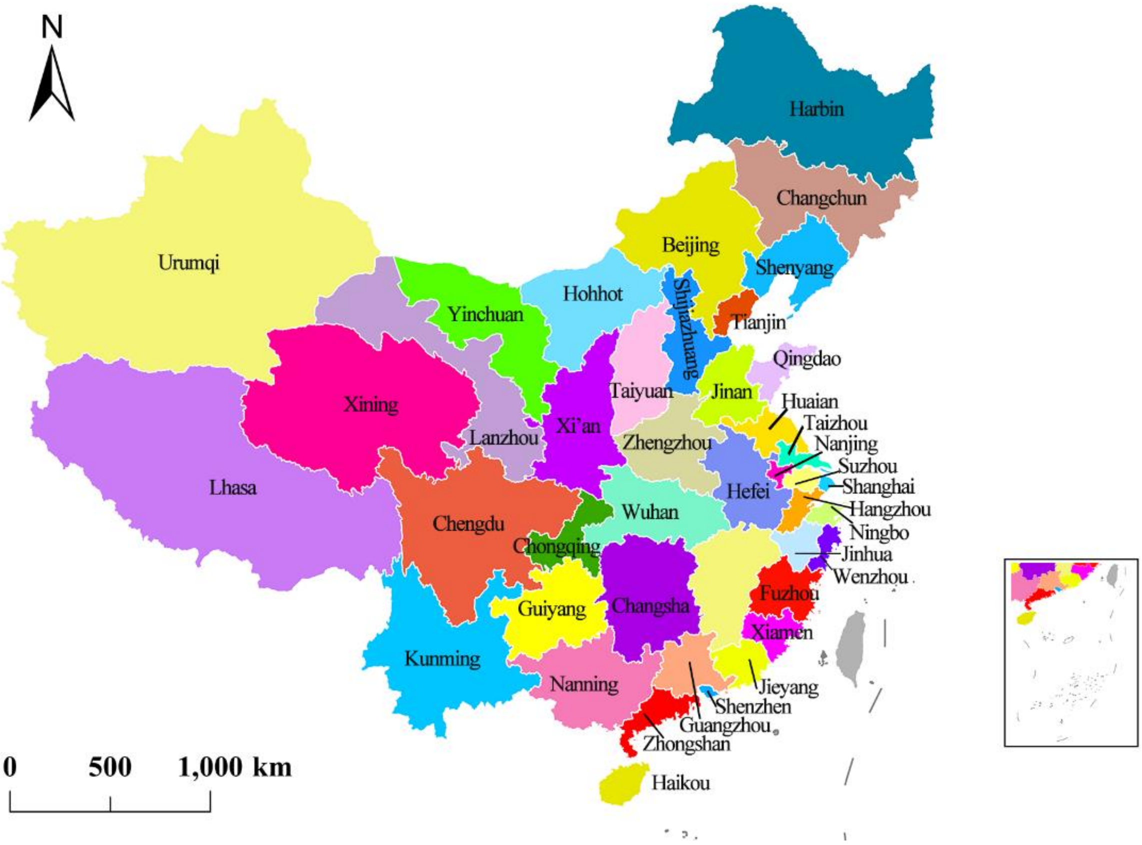

Note: this study identify 42 regional centers for inter-city express delivery according to the hub-and-spoke model and for analysis of 21,000 express delivery waybills. The coverage of each regional center was shown in figure.

Supplementary Fig.31 Spatial configuration of the regional centers in the hub and spoke model of inter-city express delivery

There existed 42 regional centers of the hub and spoke model of the Inter-city express delivery service. It was different from the provincial settings. Thereof, the developed eastern coastal provinces such as Zhejiang, Jiangsu, and Zhejiang were divided into four parts, due to the giant e-commerce companies such as Jingdong and Alibaba located in these areas. Meanwhile, Fujian province was divided into two parts, including Xiamen and Fuzhou. However, on account that Inner Mongolia spans very large, the cities of Inner Mongolia were correspondingly categorized into Harbin, Changchun, Beijing, and Yinchuan. Herein, the Guilin was brought in Changsha, while Gansu Pingliang and Qingyang are classified as xi 'an.

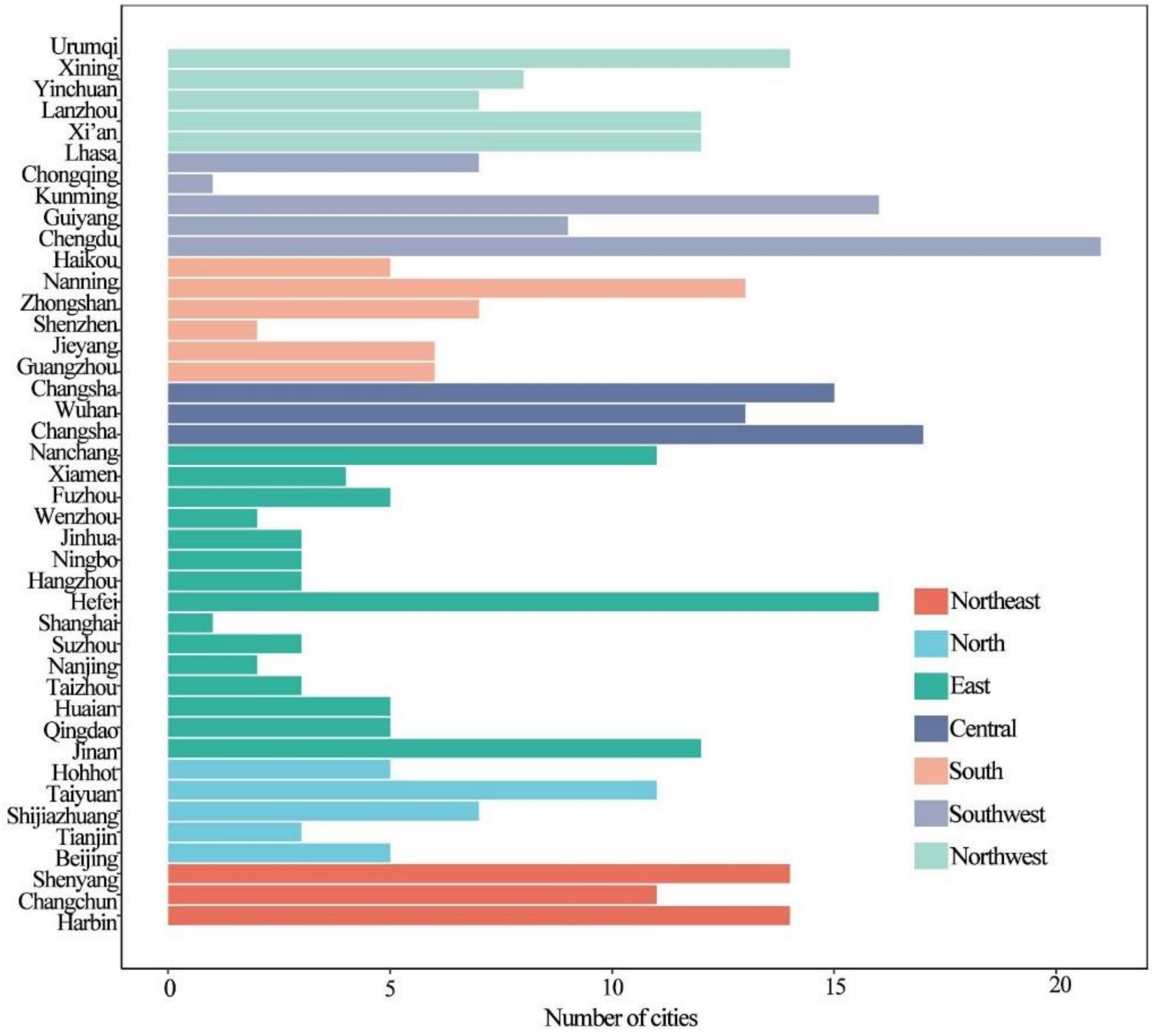

Supplementary Fig.32 Each center covering relative national cities

The covered number of cities in each regional center was ranged from 1 to 22. The cities number of the regional center in central and Northeast of China were high, while the cities number of the regional center in the east of China was low. That indicated the developed eastern coastal provinces were divided into several regional centers.

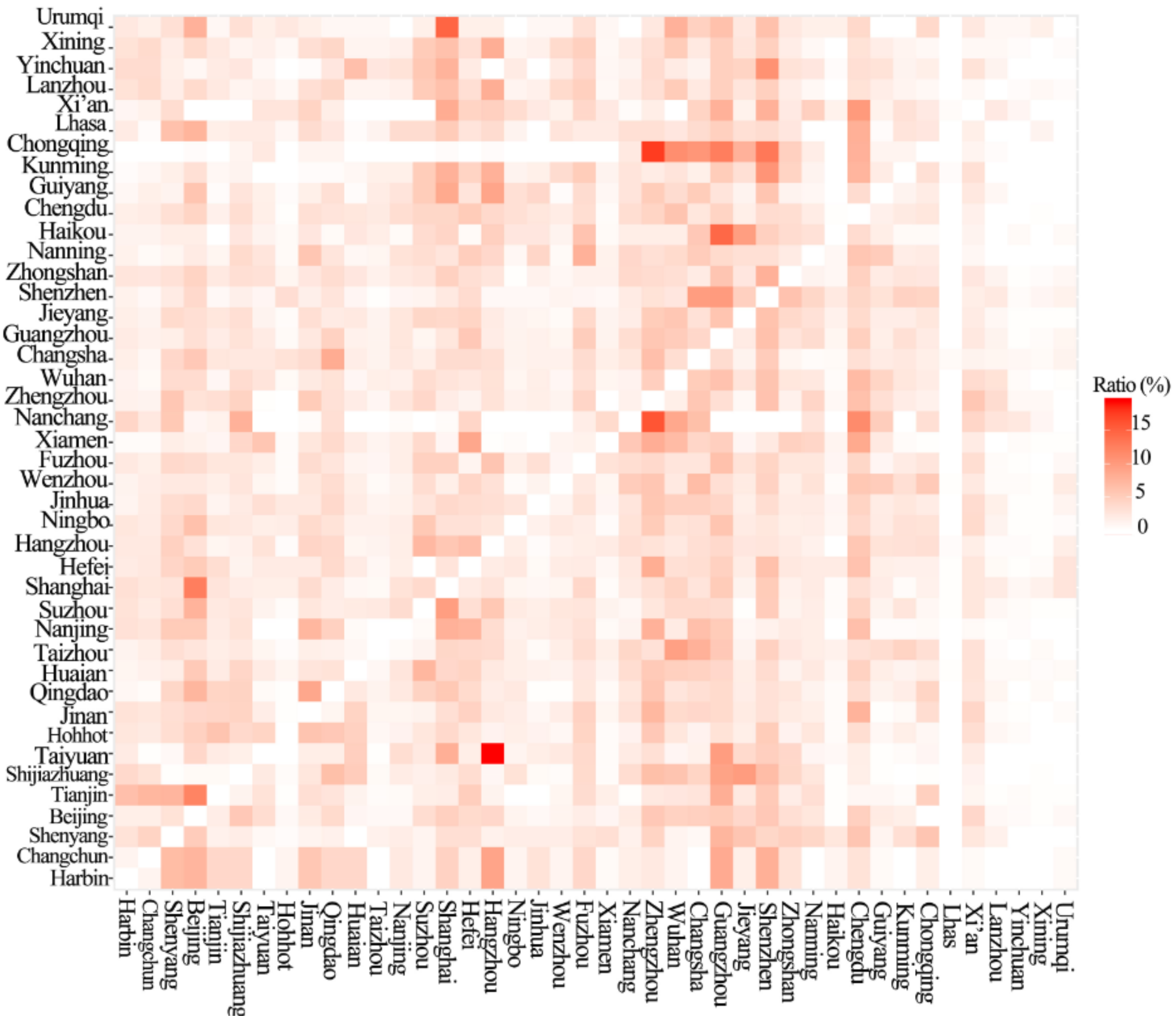

Supplementary Fig.33 The matrix of transferring ratios among 42 regional centers

(*The proportion of the total deliveries sent from a regional center to another regional center*)

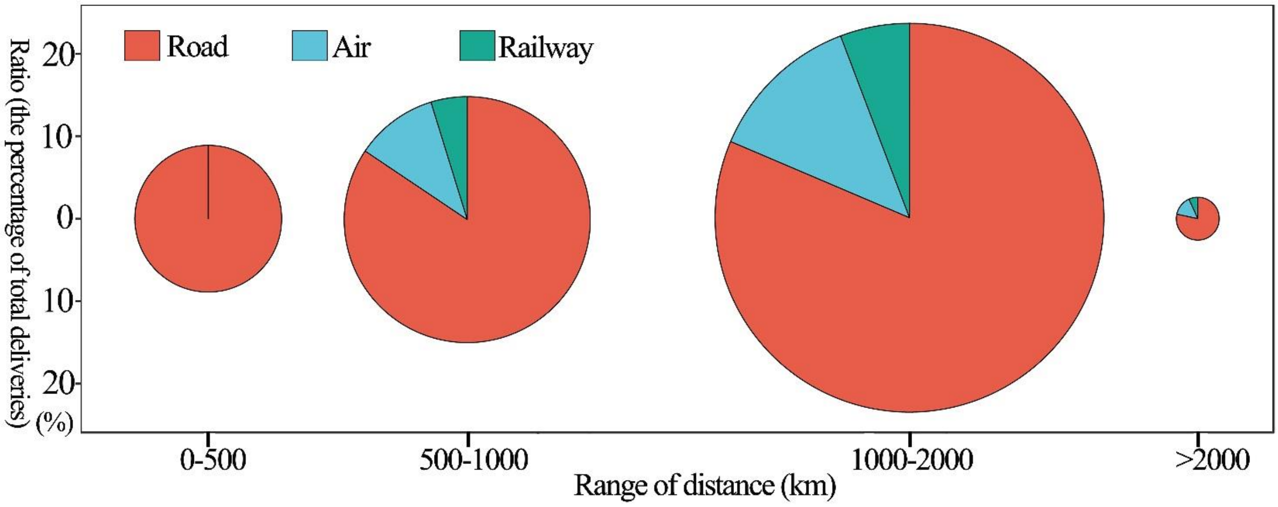

Supplementary Fig.34 Proportion of three transportation type

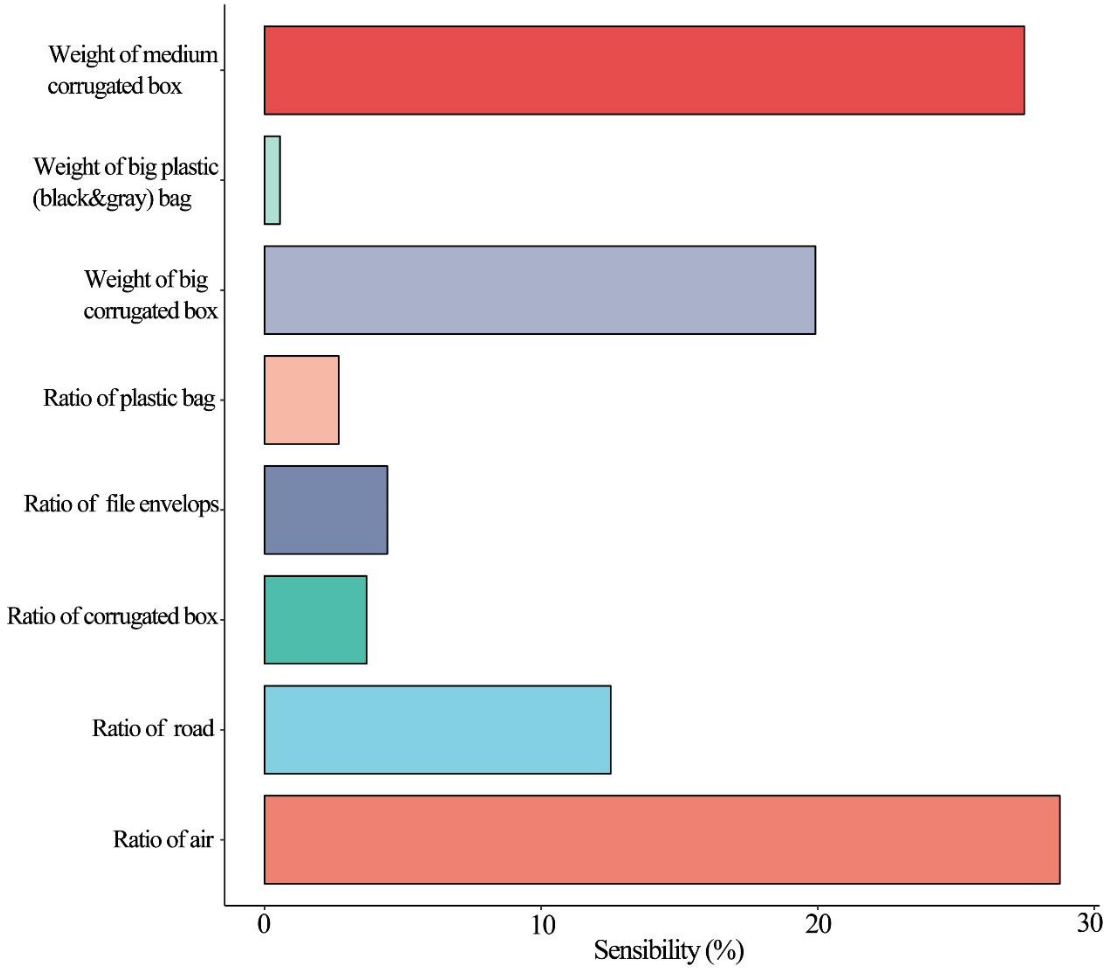

Supplementary Fig.35 Sensibility analysis of model parameter for GHG emissions

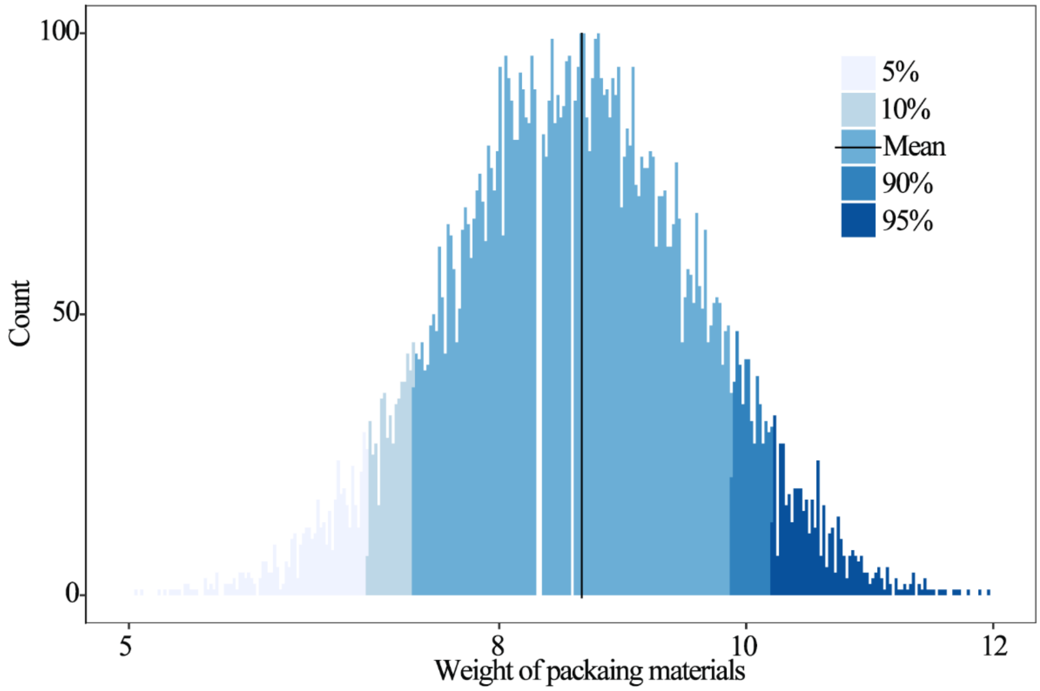

Supplementary Fig.36 Monte Carlo simulation for the total weight of scrap packaing materials

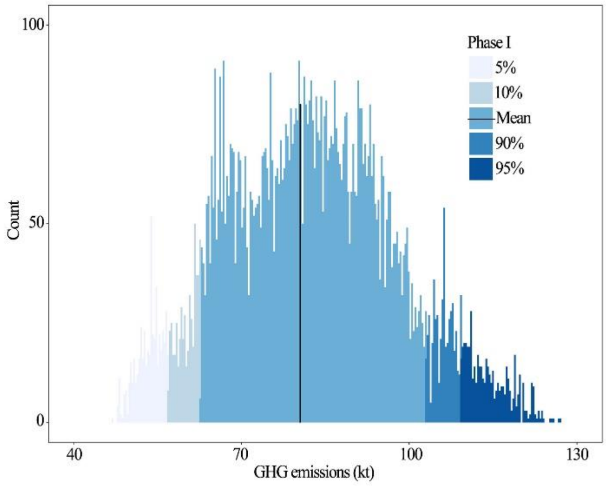

a. Phase I of inter-city express delivery

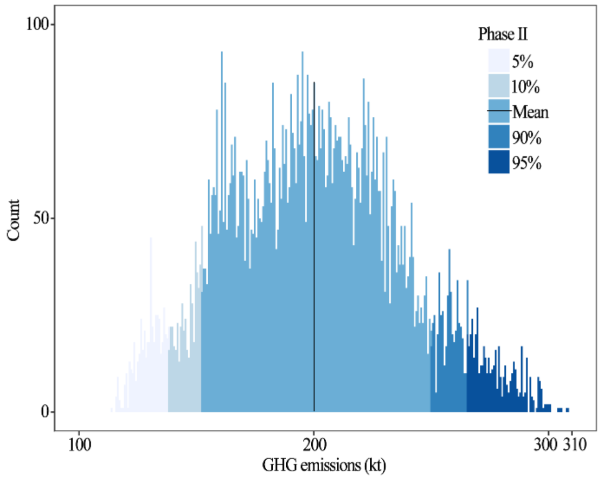

b. Phase II of inter-city express delivery

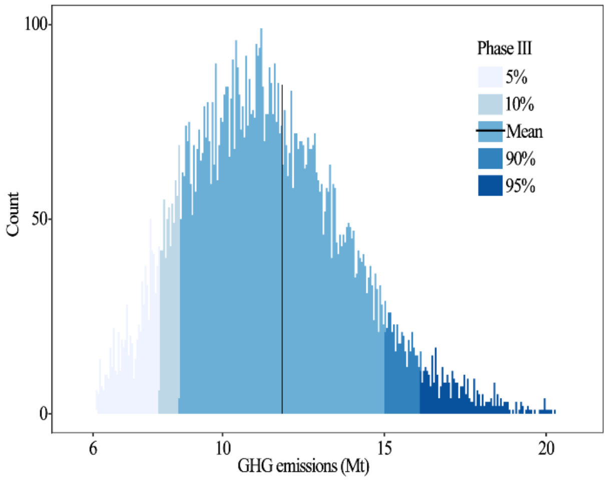

c. Phase III of inter-city express delivery

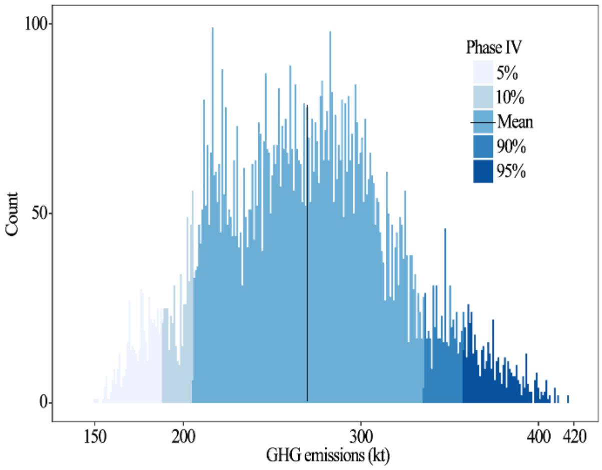

d. Phase IV of inter-city express delivery

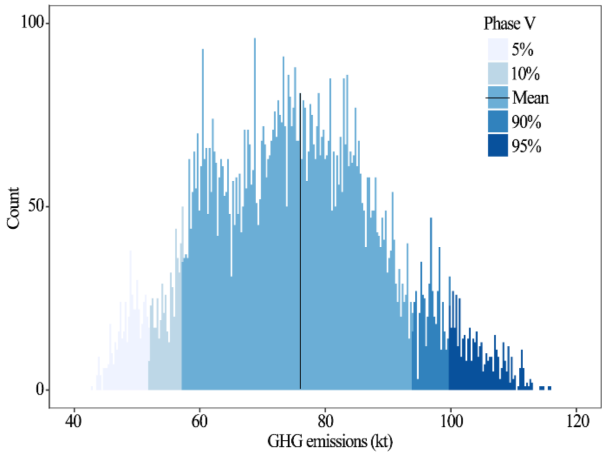

e. Phase V of inter-city express delivery

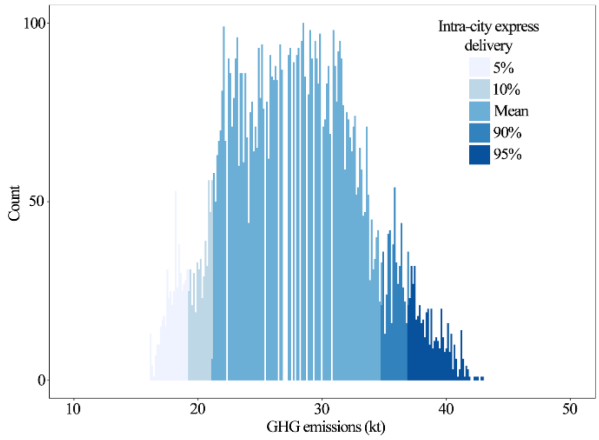

f. Intra-city express delivery

Supplementary Fig.37 Monte Carlo simulation for GHG emission from the shipment of express delivery: by distribution

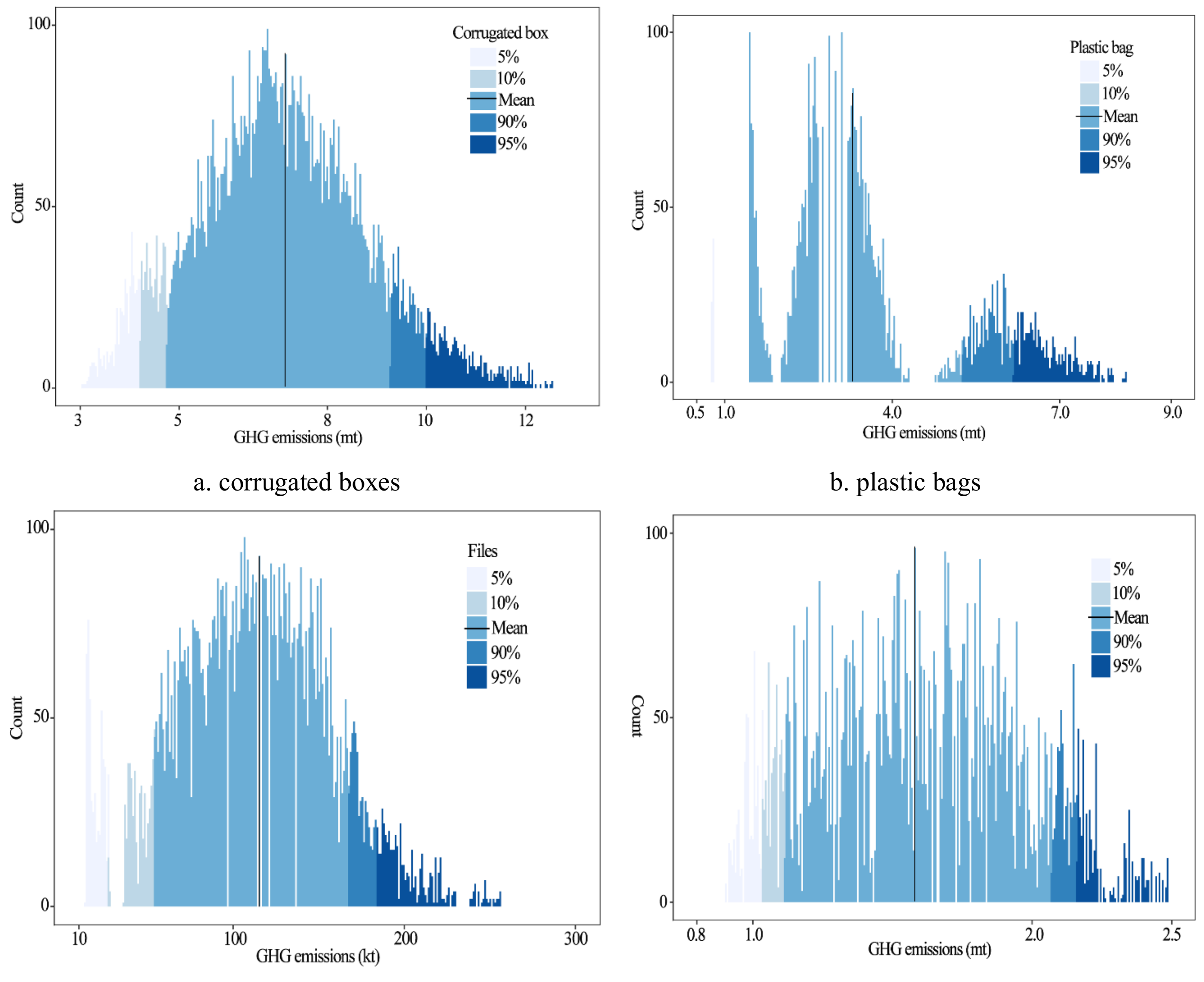

a. corrugated boxes b. plastic bags c. Files d. others package

Supplementary Fig.38 Monte Carlo simulation of GHG emission from the shipment of express delivery of four major package categories

The distribution for five phases in Inter-city and Intra-city delivery service showed the normal distribution. The mean of the phase I, II, III, IV, and V were 84, 203, 11848, 269, and 75kt. The range of deviation error of these phases was below 15%, which indicated the model parameters were reasonable and controllable.

The distribution for corrugated boxes showed the normal distribution, and the distribution for file envelopes and other packages seemingly showed the normal distribution. However, the distribution for plastic bag showed several peak distributions. The mean of GHG emission of four package types corrugated box, plastic bag, file envelopes, other packages were 7.35, 3.41, 1.6 and 0.11 Mt. The range of deviation error of these phases was below 15%, which indicated the model parameters were reasonable and controllable.

References

1 Jevinger, A. & Persson, J. A. Consignment-level allocations of carbon emissions in road freight transport. *Transport Res D-TR E.* **48**, 298–315 (2016).

2 Guajardo, M. Environmental benefits of collaboration and allocation of emissions in road freight transportation. In V. Zeimpekis, E. Aktas, M. BoOtherakis,& I. Minis (Eds.), Sustainable Freight Transport: Theory, Models, and Case Studies (2018).

3 McKinnon, A., Browne, M., Whiteing, A. & Piecyk, M. Green logistics: Improving the environmental sustainability of logistics. London: Kogan Page (2015).

4 COFRET (Carbon Footprint of Freight Transport). *Existing methods and tools for calculation of carbon footprint of transport and logistics* (2011). Available at: http://www.cofret-project.eu/downloads/pdf/COFRET_Deliverable_2.1_final.pdf

5 BSI (British Standards Institution). *PAS 2050:2011: Specification for the assessment of the life cycle greenhouse gas emissions of goods and services* (2011). Available at: http://shop.bsigroup.com/en/forms/PASs/PAS-2050/

6 CEN (European Committee for Standardization). *EN 16258:2012 - Methodology for calculation and declaration of energy consumption and GHG emissions of transport services* (freight and passengers) (2012).

7 Schmied, M. & Knörr, W. *Carbon Footprint – Teilgutachten: Monitoring fürden CO2-Ausstoß in der Logistikkette* (2012). Available at: http://www.uba.de/uba-info-medien/4306.html.

8 Davydenko, I., Ehrler, V., Ree, D. de, Lewis, A. & Tavasszy, L. Towards a global CO2 calculation standard for supply chains: suggestions for methodological improvements. *Transport Res D-TR E.* **32**, 362–372 (2014).

9 Kellner, F. Allocating greenhouse gas emissions to shipments in road freight transportation: Suggestions for a global carbon accounting standard. *Enegy Policy*. **98**, 565-575 (2016).

10 Kellner, F.& Schneiderbauer, M. Further insights into the allocation of greenhouse gas emissions to shipments in road freight transportation: The pollution routing game. *Eur J Oper Res.* **278**, 296-313 (2019).

11 Department of Electronic Commerce and Information Technology, Ministry of Commerce of China. *E commerce in China of 2018* (2019) (in Chinese)

12 State Post Bureau of China (SPBC). *The 13th Five-Year development plan for express delivery* (2017) (in Chinese).

13 State Post Bureau of China (SPBC). *Chinese Statistic Bulletin of Post Business in 2018* (2019) (in Chinese). Available at: http://www.spb.gov.cn/xw/dtxx_15079/201806/t20180604_1581131.html

14 State Post Bureau of China (SPBC). *Chinese Statistic Bulletin of Post Business in 2013* (2014) (in Chinese). Available at:

http://www.spb.gov.cn/xw/dtxx_15079/201405/t20140523_320687.html

15 Cainiao Yizhan. *Tmall double 11 logistics report* (2019) (in Chinese).

16 Bi,M., He, S.& Xu, W. Express delivery with high-speed railway: Definitely feasible or just a publicity stunt. Transport Res A-Pol. **120**, 165-187 (2019).

17 State Post Bureau of China (SPBC). *China Express Development Index Report in 2018* (2019) (in Chinese). Available at: http://www.spb.gov.cn/xw/dtxx_15079/201904/t20190417_1814716.html

18 National Development and Reform Commission (NDRC). *Announcement on Oil Products Upgrading- Announcement No.16* (2018).

19 AQSIQ (General Administration of Quality Supervision & Standardization Administration of China). *Packings for Express Service* (2018). GB/T 16606.1-2009.

20 Greenpeace. *Production characteristics and management status of express package waste in China* (2019) (in Chinese).

21 Yu, S., Yang, Z., & Yu, B. (2017). Air express network design based on express path choices – Chinese case study. *J. Air Tran. Manage*. **61**, 73–80

22 Lina,B., Zhao, Y.&Lin R. Optimization for courier delivery service network design based on frequency delay. *Comput Ind Eng*. **139**, 106114 (2019).

23 Liu, J., Huang, Y. & Chang, C. Leverage analysis of carbon market pricefluctuation in China. *J Clean Prod*. **245**, 118557 (2020).
